# microRNAs Involved in the Control of Innate Immunity in *Candida* Infected *Caenorhabditis elegans*

**DOI:** 10.1038/srep36036

**Published:** 2016-10-31

**Authors:** Lingmei Sun, Lingtong Zhi, Shumaila Shakoor, Kai Liao, Dayong Wang

**Affiliations:** 1 Key Laboratory of Developmental Genes and Human Disease in Ministry of Education, Medical School, Southeast University, Nanjing 210009, China

## Abstract

The role of microRNAs (miRNAs) in regulating innate immune response to *Candida albicans* infection in *Caenorhabditis elegans* is still largely unclear. Using small RNA SOLiD deep sequencing technique, we profiled the miRNAs that were dysregulated by *C. albicans* infection. We identified 16 miRNAs that were up-regulated and 4 miRNAs that were down-regulated in nematodes infected with *C. albicans*. Bioinformatics analysis implied that these dysregulated miRNAs may be involved in the control of many important biological processes. Using available mutants, we observed that *mir-251* and *mir-252* loss-of-function mutants were resistant to *C. albicans* infection, whereas *mir-360* mutants were hypersensitive to *C. albicans* infection. The expression pattern of antimicrobial genes suggested that *mir-251*, *mir-252*, and *mir-360* played crucial roles in regulating the innate immune response to *C. albicans* infection. Fungal burden might be closely associated with altered lifespan and innate immune response in *mir-251*, *mir-252*, and *mir-360* mutants. Moreover, *mir-251* and *mir-252* might function downstream of p38 mitogen activated protein kinase (MAPK) or IGF-1/insulin-like pathway to regulate the innate immune response to *C. albicans* infection. Our results provide an important molecular basis for further elucidating how miRNA-mRNA networks may control the innate immune response to *C. albicans* infection.

*Candida albicans* is the most common fungal pathogen for human beings^1^. Under most circumstances, *C. albicans* is harmless. However, it can invade host tissues and cause life-threatening infections when the immune system is weakened (e.g. from critical illness) and the competing bacterial flora are eliminated (e.g. from broad-spectrum antibiotic use)^2^,^3^,^4^.

Innate immune system, the first line of defense against environmental microbial infection, is evolutionarily conserved between vertebrate and invertebrate animals^5^,^6^. Innate immunity, induced by phagocytosis or production of antimicrobial peptides, has been detected in a wide variety of animals, which suggests that we can use certain invertebrate model animals to elucidate the cellular and molecular mechanisms of innate immunity^5^,^7^. After infection with pathogens, the nematode *Caenorhabditis elegans* can exhibit a rapid innate immune response, and produce an array of anti-microbial proteins, as observed in other organisms in the animal kingdom^8^,^9^. *C. elegans* is considered to have an innate immune defense system, due to its expression of antimicrobial peptides to defend against pathogen infection^8^,^10^. *C. elegans* has been proven to be helpful for the study of virulence of human pathogenic fungi, such as *C. albicans*^11^. This model organism can provide useful insights into the mechanisms underlying fungal virulence and host immunity^11^,^12^. Some signaling pathways, such as the p38 mitogen activated protein kinase (MAPK) cascade, have been found to combat the fungal invasion^11^,^12^. Moreover, genome-wide transcriptional profiling indicates that infection of *C. elegans* with *C. albicans* yeast induces bidirectional changes in expression of specific genes, including antimicrobial, secreted, and detoxification proteins^13^.

MicroRNAs (miRNAs) are pivotal regulators for gene expression in metazoa^14^. In animals, miRNAs normally target several mRNAs through base pairing with 3′-untranslated region (3′-UTR) of the corresponding target mRNAs^15^. miRNAs have been shown to be involved in various biological processes, including patterning of the nervous system, inflammation and immunity, cell death and proliferation, and development^15^,^16^. In recent years, some evidence has demonstrated that miRNAs may play important roles in the regulation of innate immunity^17^,^18^. In *C. elegans*, some specific miRNAs have been shown to be involved in the control of innate immune response to bacterial pathogens^19^,^20^,^21^. However, the role of miRNAs in the control of the *C. elegans* innate immune response to *C. albicans* infection is still unclear.

To better understand the roles of miRNAs in the control of innate immunity in *C. albicans* infected *C. elegans*, herein, we profiled miRNA expression in nematodes infected with *C. albicans* SC5314 using the technique of small RNA deep sequencing. We confirmed the functions of some candidate miRNAs that regulate *C. albicans* infection using the available miRNAs mutants. Our data suggest a crucial role of some miRNAs in regulating the innate immune response to pathogenic fungi. Our study provides an important basis for further elucidating the molecular mechanisms of the innate immune response of nematodes to *C. albicans* infection.

## Results

### Intact *C. albicans* cells accumulated within the body of *C. elegans*

*C. elegans* can eat microorganisms, but die when fed with certain pathogens, such as the *C. albicans*^5^,^7^,^13^. In this study, we first used a *C. albicans* reporter strain (CaSA1 expressing yeast-enhanced GFP) to examine the persistence of *C. albicans* cells within the pharynx and the intestine of *C. elegans*. After feeding *C. albicans* to nematodes, the intact yeast cells severely infected the pharyngeal grinder organ, as well as the proximal, middle, and distal intestine (Fig. S1). In contrast, *E. coli* OP50 cells, which are normally non-pathogenic to nematodes, were seldom accumulated in the pharynx and intestine (Fig. S1). In addition, only a very small amount of heat-killed *C. albicans* was observed in the pharynx and intestine in nematodes (Fig. S1).

### RNAomics assay validation

To determine the possible influence of *C. albicans* SC5314 infection on miRNAs, we used SOLiD sequencing to analyze miRNA expression. RNA profiling was performed to compare miRNA expression profiles between control (treatment with heat-killed *C. albicans* SC5314) and *C. albicans* SC5314 infection conditions. Comparison of the number of colony-forming units (CFU) between live SC5314 and heat-killed SC5314 in nematodes suggests that heat-killing effectively prevents *C. albicans* SC5314 infection (Fig. S2). We performed cluster analysis according to length of the detected miRNA sequences. Most of the detected miRNA sequences were 21–24 nucleotides, which were considered to be mature miRNAs by subsequent miRNA database blasting (Fig. S3a). Next, we analyzed the chromosomal distribution of the detected miRNA sequences. The miRNAs detected by SOLiD sequencing were located on all chromosomes, including the sex chromosome X, and most detected miRNAs were located on chromosomes II, IV, and X (Fig. S3b). These results imply that RNAomics analysis is a valid approach to detect dysregulated miRNAs induced by *C. albicans* infection.

### Dysregulated miRNA expression in *C. albicans* infected *C. elegans*

**Figure 1:**
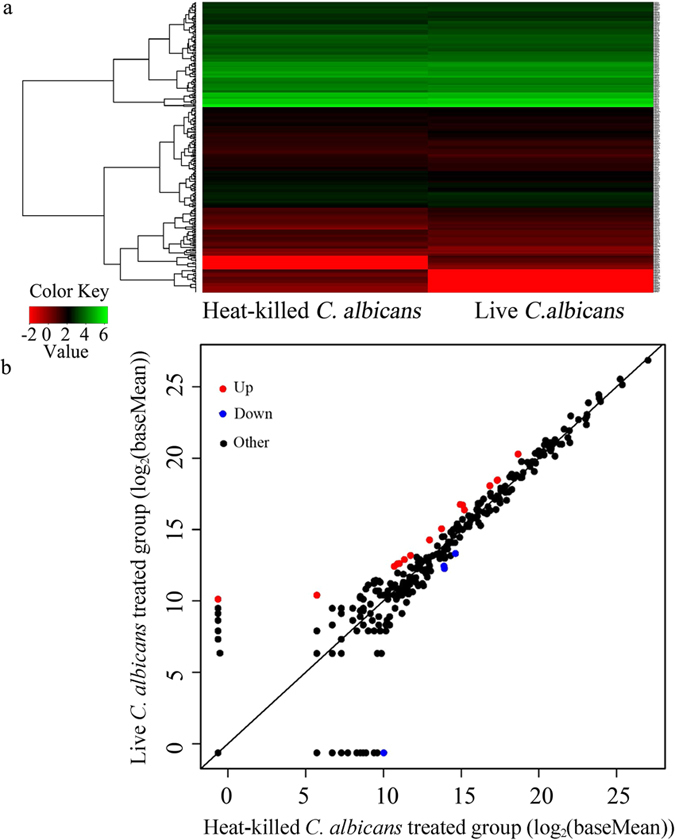
Results of SOLiD sequencing. (**a**) miRNAs expression analysis by hierarchical clustering assay to reveal a characteristic molecular signature for *C. albicans* SC5314 infected *C. elegans*. (**b**) Scatter diagram of miRNAs in *C. albicans* treated *C. elegans*.

**Figure 2:**
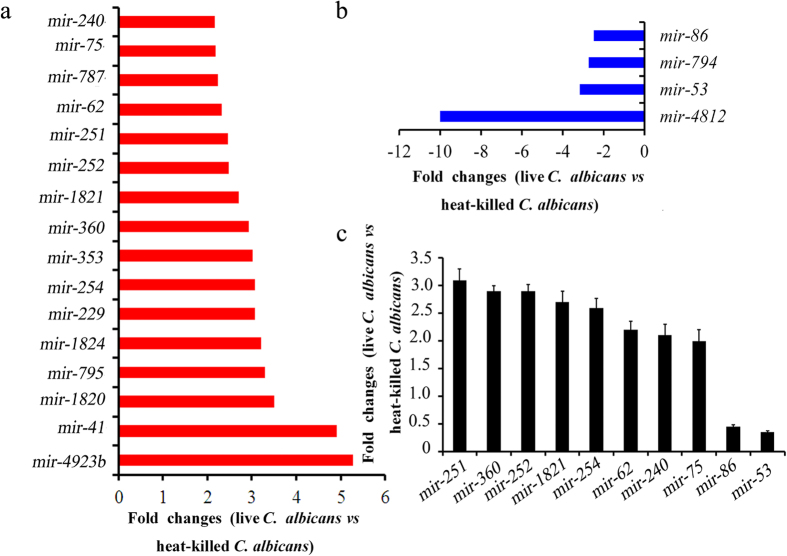
miRNAs expression in *C. albicans* infected *C. elegans*. (**a**) Fold changes of upregulated miRNAs in *C. albicans* SC5314 infected *C. elegans*. (**b**) Fold changes of downregulated miRNAs in *C. albicans* SC5314 infected *C. elegans*. (**c**) Expression pattern of mature miRNAs detected by qRT-PCR. Bars represent means ± S.E.M.

After SOLiD sequencing, we compared miRNA expression profiles between control and *C. albicans* SC5314 infection conditions. Dysregulated miRNAs in *C. albicans* SC5314 infected *C. elegans* were identified using statistical significance and a 2.0 fold-change as cutoff criteria. We obtained the annotations of differentially expressed miRNAs by comparing our detected miRNA sequences (Table S1) with Genbank and miRbase databases (Fig. 1a). We ultimately identified 20 differentially expressed miRNAs in *C. albicans* SC5314 infected *C. elegans* compared with control (Table S2). Among these miRNAs, 16 were up-regulated and 4 were down-regulated following *C. albicans* SC5314 infection (Fig. 1b, Table S2). The up-regulated miRNAs included *mir-240*, *mir-75*, *mir-787*, *mir-62*, *mir-251*, *mir-252*, *mir-1821*, *mir-360*, *mir-353*, *mir-254*, *mir-229*, *mir-1824*, *mir-795*, *mir-1820*, *mir-41*, and *mir-4923b*, while the down-regulated miRNAs included *mir-4812*, *mir-53*, *mir-794*, and *mir-86* in *C. albicans* SC5314 infected nematodes (Fig. 2a,b).

### Confirmation of dysregulated miRNAs in *C. albicans* infected *C. elegans*

We further selected several candidate miRNAs, and verified our sequencing results using quantitative real-time polymerase chain reaction (qRT-PCR). After infection with *C. albicans*, qRT-PCR assays showed that the expression levels of *mir-251*, *mir-360*, *mir-252*, *mir-1821*, *mir-254*, *mir-62*, *mir-240*, and *mir-75* were significantly increased, and the expression levels of *mir-86* and *mir-53* were significantly decreased (Fig. 2c). Thus, the expression patterns for these candidate miRNAs were similar to those from the SOLiD sequencing.

### Prediction of dysregulated miRNAs’ target genes and gene ontology assessment

**Figure 3:**
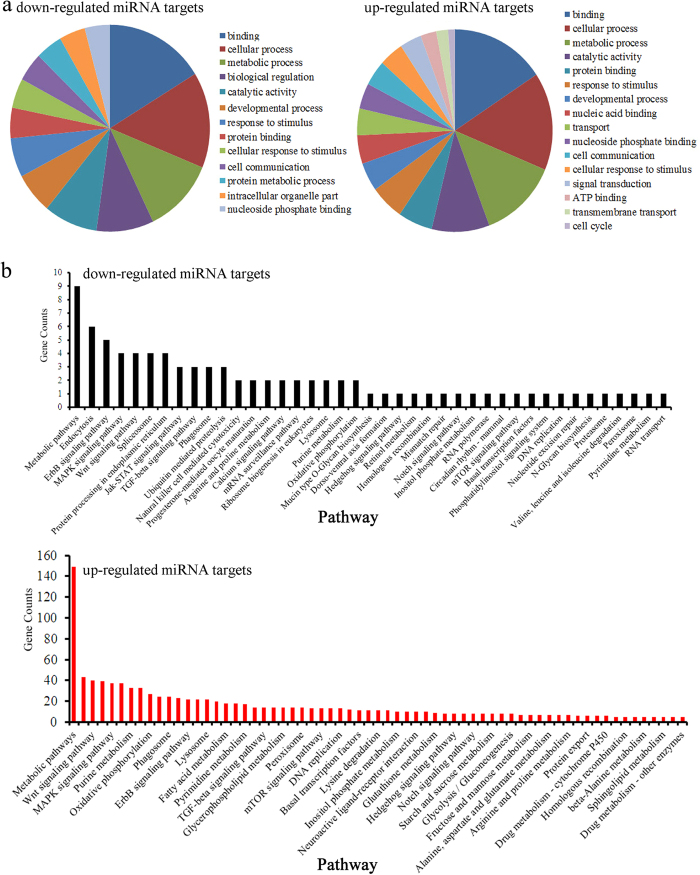
Assessment of gene ontology terms and signaling pathways. (**a**) Gene ontology terms based on dysregulated miRNAs in *C. albicans* SC5314 infected *C. elegans*. (**b**) Predicted KEGG signaling pathways based on dysregulated miRNAs in *C. albicans* SC5314 infected *C. elegans*.

Using the TargetScan database, we predicted the potential targeted genes for the detected dysregulated miRNAs. Gene ontology analysis can provide the ontology for defined terms and describe the gene product properties^22^. Based on the list of dysregulated miRNAs and their predicted target genes, we further used DESeq data to determine the possibly affected biological processes by *C. albicans* SC5314 infection. Our data showed that 62 down-regulated and 58 up-regulated gene ontology terms were possibly associated with the control of *C. albicans* SC5314 infection (Tables S3 and S4). The significantly altered gene ontology terms were classified into several categories, which contained the following biological processes: development, intracellular organelle, cell cycle, cellular transportation, signal transduction, protein binding, cellular metabolism, cell communication, and response to stimulus (Fig. 3a).

### Analysis of signaling pathways mediated by the predicted target genes for dysregulated miRNAs in *C. albicans* infected *C. elegans*

We also employed the Kyoto Encyclopedia of Genes and Genomes (KEGG) pathway database to determine if the predicted target genes of dysregulated miRNAs belong to particular signaling pathways. KEGG pathway mapping can be used to map molecular datasets, especially large-scale genomics datasets, and the relevant signaling pathways can be further extracted using its pathway mining tool^23^. We identified 41 potential signaling pathways for the down-regulated miRNAs and 108 potential signaling pathways for the up-regulated miRNAs (Tables S5 and S6). The signaling pathways hypothesized to be affected by *C. albicans* infection mainly included signaling pathways related to development, reproduction, cellular metabolism, cellular transportation, endocytosis, phagosome, proteasome, peroxisome and lysosome, protein processing in the endoplasmic reticulum, ubiquitin mediated proteolysis, and circadian rhythm (Fig. 3b). The signaling pathways influenced by *C. albicans* infection also contained several developmental and biochemical signaling pathways, such as the Wnt, Hedgehog, Notch, MAPK, mTOR, ErbB, Jak-STAT, TGF-beta, and calcium signaling pathways, and the mRNA surveillance pathway (Fig. 3b).

### Effect of candidate miRNAs on *C. elegans* lifespan after *C. albicans* infection

**Figure 4:**
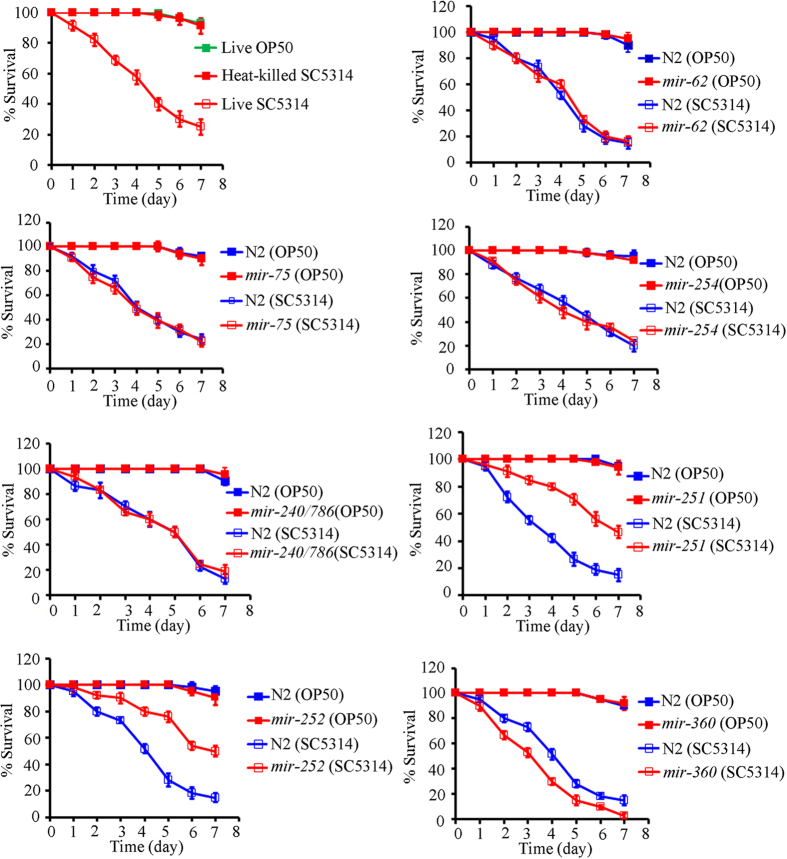
Lifespan in mutants of candidate miRNA infected with *C. albicans*. Sixty nematodes were examined per treatment. Comparisons of survival plots between wild-type N2 and mutant infected with *C. albicans* SC5314 were performed. After *C. albicans* SC5314 infection, statistical comparisons of the survival plots indicate that survival of the *mir-251*, *mir-252*, or *mir-360* mutant was significantly different from that in wild-type N2 (*P* < 0.0001). In contrast, after *C. albicans* SC5314 infection, statistical comparisons of the survival plots indicate that survival of the *mir-62* (*P* = 0.9734), *mir-75* (*P* = 0.9812), *mir-254* (*P* = 0.9241), or *mir-240/786* (*P* = 0.9318) was not significantly different from that in wild-type N2.

To understand the function of our candidate miRNAs in in *C. elegans’* immune response to *C. albicans* infection, we employed the available miRNA mutants (*mir-251*(*n4606*), *mir-252*(*n4570*), *mir-254*(*n4470*), *mir-360*(*n4635*), *mir-240/786*(*n4541*), *mir-62*(*n4539*), and *mir-75*(*n4472*)) to examine the effects of loss-of-function mutations of candidate miRNAs on *C. elegans* lifespan after *C. albicans* SC5314 infection. In wild-type nematodes, lifespan was noticeably reduced by feeding with live *C. albicans* SC5314, compared to feeding with *E. coli* OP50 or heat-killed *C. albicans* SC5314 (Fig. 4). Among the miRNA mutants we examined, we found that the *mir-251*(*n4606*) and *mir-252*(*n4570*) mutants were resistant to *C. albicans* SC5314 infection. *C. albicans* SC5314 infected *mir-251*(*n4606*) and *mir-252*(*n4570*) mutant showed the increased lifespan compared with *C. albicans* SC5314 infected wild-type N2 nematodes (Fig. 4). In contrast, the *mir-360*(*n4635*) mutant was hypersensitive to *C. albicans* SC5314 infection. *C. albicans* SC5314 infected *mir-360*(*n4635*) mutant showed the reduced lifespan compared with *C. albicans* SC5314 infected wild-type N2 nematodes (Fig. 4). Mutations in *mir-62*, *mir-75*, *mir-254*, or *mir-240/786* did not noticeably influence lifespan after *C. albicans* SC5314 infection (Fig. 4). Under normal conditions, the *mir-251*(*n4606*), *mir-252*(*n4570*), and *mir-360*(*n4635*) mutants had similar lifespan to wild-type N2 nematodes (Fig. S4).

### Candidate miRNA mutants’ innate immune response to *C. albicans* infection

**Figure 5:**
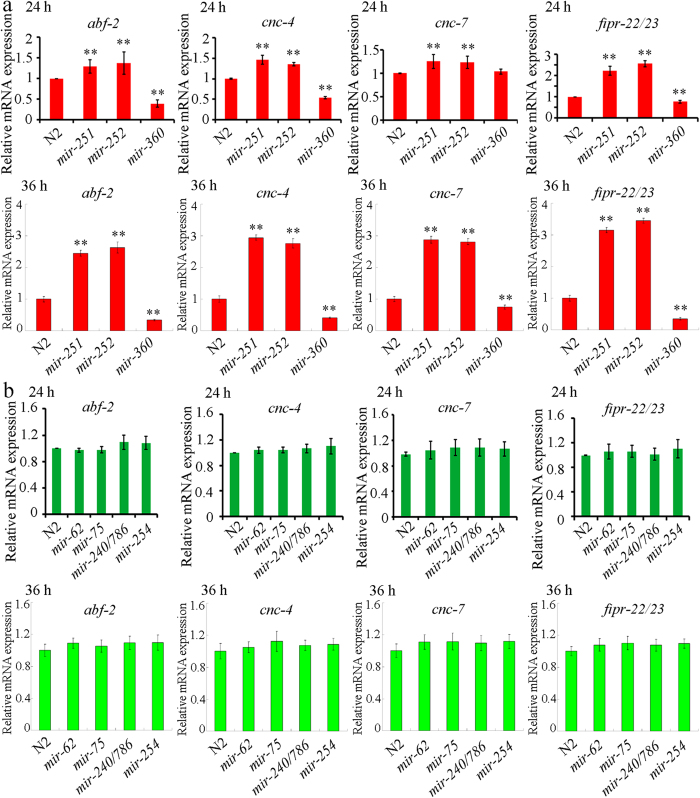
Expression patterns of antimicrobial genes in *C. albicans* infected *C. elegans* based on qRT-PCR analysis. (**a**) Expression patterns of antimicrobial genes in *mir-251*, *mir-252*, or *mir-360* mutant infected with *C. albicans* SC5314 for 24 or 36 h. (**b**) Expression patterns of antimicrobial genes in *mir-62*, *mir-75*, *mir-240/786*, or *mir-254* mutant infected with *C. albicans* SC5314 for 24 or 36 h. The data are presented as the average of three biological replicates each normalized to control *act-1* gene. Bars represent means ± S.E.M. ^**^P < 0.01 *vs* N2.

In response to *C. albicans* infection, *C. elegans* will produce an array of antimicrobial proteins^13^. To understand the role our candidate miRNAs in regulating the innate immune response, we investigated the expression patterns of some antimicrobial genes (*abf-2*, *cnc-4*, *cnc-7*, and *fipr-22/33*) in candidate miRNA mutants after infection with *C. albicans* SC5314 for 24 or 36 h. The antimicrobial genes associated with the innate immune response to *C. albicans* infection were selected based on previous findings^13^. Infection with *C. albicans* SC5314 increased the mRNA expression of *abf-2*, *cnc-4*, *cnc-7*, and *fipr-22/33* in wild-type N2 nematodes^13^. After infection with *C. albicans* SC5314 for 24 or 36 h, loss-of-function mutation of *mir-251* or *mir-252* caused the increased expression levels of *abf-2*, *cnc-4*, *cnc-7*, and *fipr-22/23* compared with wild-type N2 nematodes (Fig. 5a). In contrast, after infection with *C. albicans* SC5314 for 24 h, loss-of-function mutation of *mir-360* resulted in the decreased expression levels of *abf-2*, *cnc-4*, and *fipr-22/23* compared with wild-type N2 nematodes (Fig. 5a). After infection with *C. albicans* SC5314 for 36 h, loss-of-function mutation of *mir-360* further caused the decrease in expression levels of *abf-2*, *cnc-4*, *cnc-7*, and *fipr-22/23* relative to wild-type N2 nematodes (Fig. 5a). Meanwhile, after infection with *C. albicans* SC5314 for 24 or 36 h, loss-of-function mutation of *mir-62*, *mir-75*, *mir-240/786*, or *mir-254* did not significantly affect the expression of *abf-2*, *cnc-4*, *cnc-7*, and *fipr-22/23* compared with wild-type N2 nematodes (Fig. 5b). These results suggest that *mir-251*, *mir-252*, and *mir-360* possibly play crucial roles in regulating the innate immune response of nematodes to *C. albicans* SC5314 infection.

### *C. albicans* colony formation in infected candidate miRNA mutants

**Figure 6:**
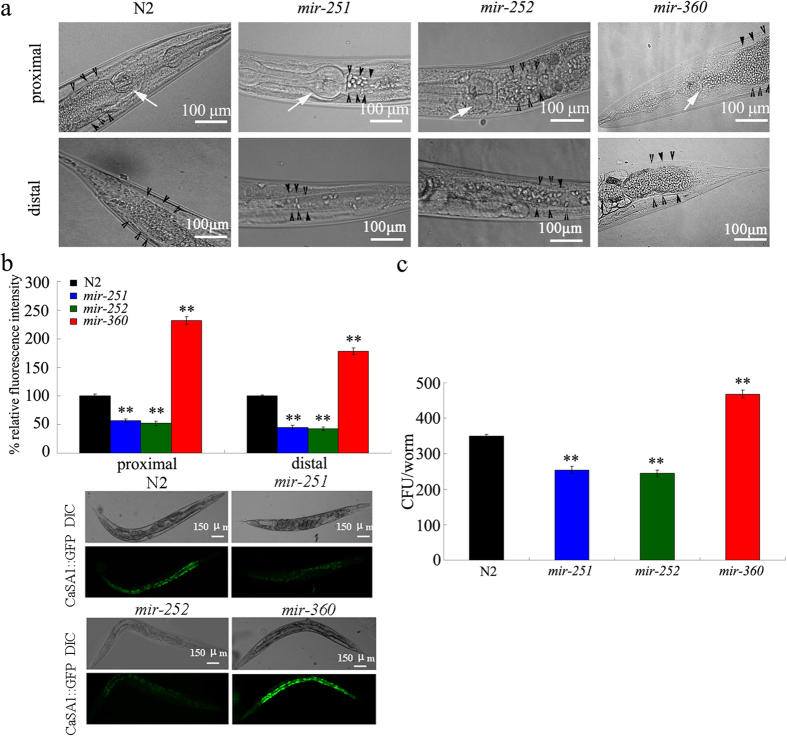
Colony forming in mutants of candidate miRNAs infected with *C. albicans.* (**a**) Shown are micrographs of wild-type N2 and *mir-251*, *mir-252*, and *mir-360* mutants infected with *C. albicans* SC5314 for 16 h. White arrowheads indicate the pharynx, and black arrows indicate the accumulation of *C. albicans* in proximal or distal of the intestine in *C. elegans*. (**b**) Comparison of relative fluorescent intensity of CaSA1::GFP in intestine of nematodes. *C. albicans* strain of CaSA1 was fed for 16 h. (**c**) Comparison of colony-forming units (CFU) between wild-type N2 and *mir-251*, *mir-252*, or *mir-360* mutant. Live *C. albicans* SC5314 was recovered from *C. elegans* after 24 h of infection. Bars represent means ± S.E.M. ^**^P < 0.01 *vs* N2.

We further investigated the fungal burden in the intestine of candidate miRNA mutants. After infection with *C. albicans* SC5314 for 16 h, mutation of *mir-251* or *mir-252* reduced the amount of *C. albicans* cells in both the proximal and distal intestine in nematodes. Conversely, mutation of *mir-360* exacerbated the accumulation of *C. albicans* cells in both the proximal and distal intestine in nematodes (Fig. 6a). In addition, after infection with *C. albicans* SC5314 for 16 h, *mir-251* and *mir-252* mutants had less distended intestines compared with wild-type N2; however, *mir-360* mutants had more distended intestines compared with wild-type N2 (Fig. 6a). Using the *C. albicans* CaSA1 strain, which expresses *GFP* under the control of the *CDR1* promoter^24^, we further observed that mutation of *mir-251* or *mir-252* reduced the relative fluorescence intensity of CaSA1::GFP in both the proximal and distal intestine in nematodes (Fig. 6b). In contrast, mutation of *mir-360* increased the relative fluorescence intensity of CaSA1::GFP in both the proximal and the distal intestines in nematodes (Fig. 6b). After infection for 24 h, wild-type *C. elegans* had an average of 361.7 colony-forming units (CFU) per worm (Fig. 6c). In contrast, after infection for 24 h, *mir-251* and *mir-252* mutations significantly reduced the CFU per worm, whereas *mir-360* mutation significantly increased the CFU per worm (Fig. 6c). Therefore, *mir-251*, *mir-252*, and *mir-360* may alter *C. albicans* colony formation in the intestine of nematodes.

### *mir-251* or *mir-252* functioned downstream of p38 MAPK or IGF-1/insulin-like pathway to regulate the innate immunity in *C. albicans* infected nematodes

**Figure 7:**
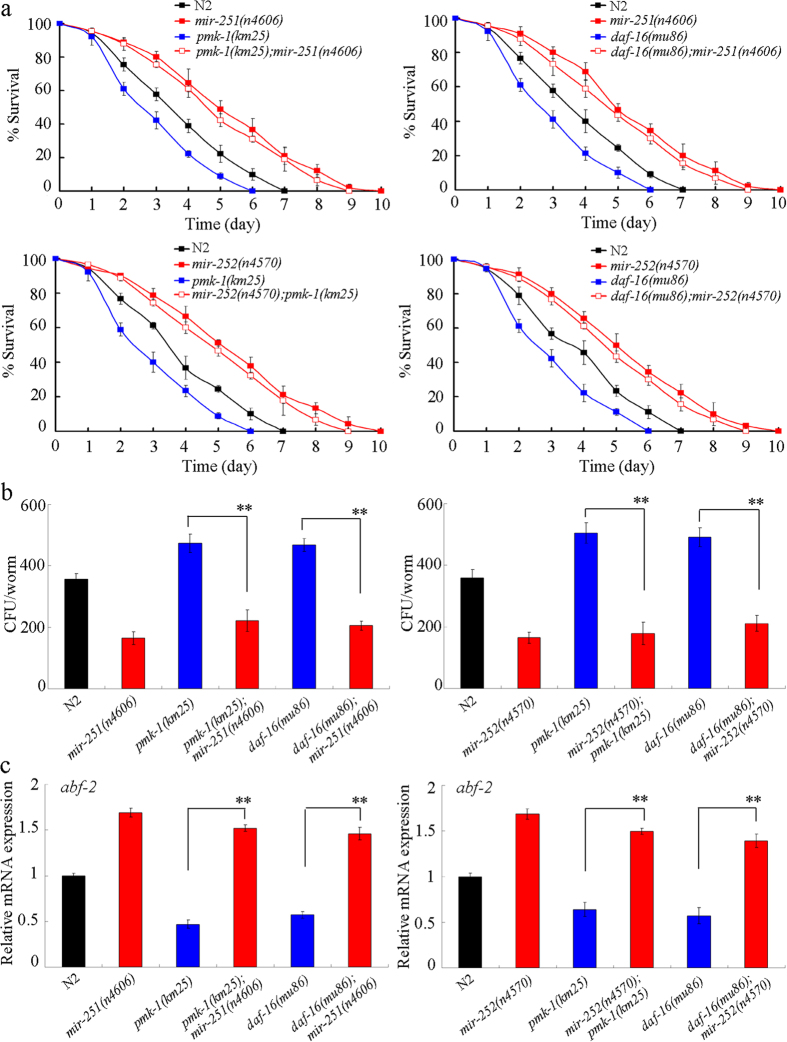
Genetic interaction of *mir-251* or *mir-252* with *pmk-1* or *daf-16* in regulating innate immunity in *C. albicans* infected nematodes. (**a**) Genetic interaction of *mir-251* or *mir-252* with *pmk-1* or *daf-16* in regulating lifespan in nematodes infected with *C. albicans* for 24 h. After *C. albicans* SC5314 infection, statistical comparisons of the survival plots indicate that survival of the *pmk-1*(*km25*)*; mir-251*(*n4606*) or *mir-252*(*n4570*)*; pmk-1*(*km25*) was significantly (*P* < 0.0001) different from that in *pmk-1*(*km25*) animals, and the *daf-16*(*mu86*)*; mir-251*(*n4606*) or *daf-16*(*mu86*)*; mir-252*(*n4570*) was significantly (*P* < 0.0001) different from that in *daf-16*(*mu86*) animals. (**b**) Genetic interaction of *mir-251* or *mir-252* with *pmk-1* or *daf-16* in regulating SC5314 CFU in *C. albicans* infected nematodes. Live *C. albicans* SC5314 were recovered from *C. elegans* after 24 h of infection. (**c**) Genetic interaction of *mir-251* or *mir-252* with *pmk-1* or *daf-16* in regulating *abf-2* expression in nematodes infected with *C. albicans* for 24 h. Bars represent means ± S.E.M. ^**^P < 0.01.

In order to further understand the molecular basis of the candidate miRNAs’ roles in innate immunity, we next examined the genetic interaction between two miRNAs (*mir-251* and *mir-252*) and p38 MAPK signaling or IGF-1/insulin-like signaling. In *C. elegans*, both the p38 MAPK signaling pathway and the IGF-1/insulin-like signaling pathway are well known to play important roles in regulating innate immunity^8^,^10^,^11^. In *C. elegans*, *pmk-1* encodes a MAP kinase (MAPK) in the p38 MAPK signaling pathway, and *daf-16* encodes a FOXO transcription factor in the IGF-1/insulin-like signaling pathway. After *C. albicans* SC5314 infection, we found that loss-of-function of *mir-251* or *mir-252* suppressed the susceptibility of *pmk-1*(*km25*) mutant nematodes to *C. albicans* infection and decreased the SC5314 CFU in *C. albicans* infected *pmk-1*(*km25*) mutant nematodes (Fig. 7a,b). Similarly, loss-of-function of *mir-251* or *mir-252* inhibited *daf-16*(*mu86*) mutants’ susceptibility to *C. albicans* infection and suppressed *C. albicans* SC5314 colony formation in *daf-16*(*mu86*) mutant nematodes (Fig. 7a,b). Moreover, loss-of-function of *mir-251* or *mir-252* significantly increased the transcriptional expression of *abf-2* in *C. albicans* infected *pmk-1*(*km25*) mutant nematodes, and loss-of-function of *mir-251* or *mir-252* also significantly increased transcriptional expression of *abf-2* in *C. albicans* infected *daf-16*(*mu86*) mutant nematodes (Fig. 7c). Therefore, our results suggest that *mir-251* and *mir-252* may function downstream of the p38 MAPK or IGF-1/insulin-like pathway to regulate the innate immune response of nematodes to *C. albicans* infection.

### Genetic interaction between *mir-251* and *mir-252* in the regulation of innate immunity in *C. albicans* infected nematodes

**Figure 8:**
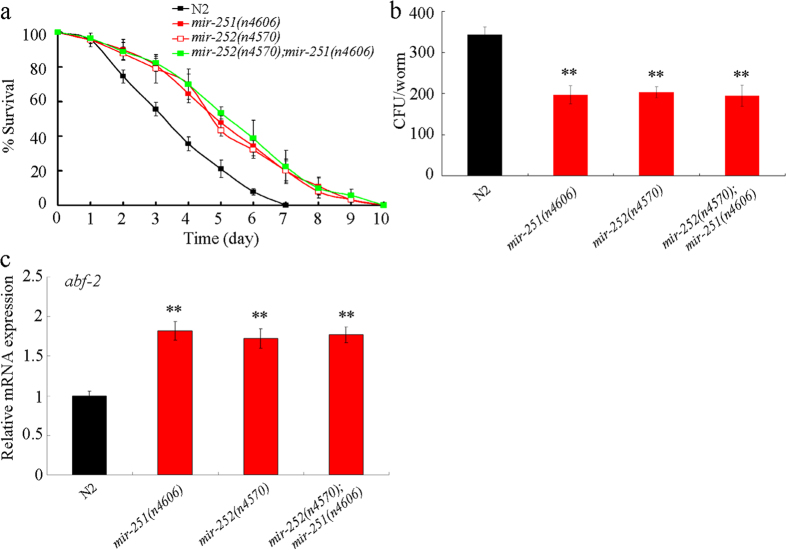
Genetic interaction between *mir-251* and *mir-252* in regulating innate immunity in *C. albicans* infected nematodes. (**a**) Genetic interaction between *mir-251* and *mir-252* in regulating lifespan in nematodes infected with *C. albicans* for 24 h. After *C. albicans* SC5314 infection, statistical comparison of the survival plots indicate that survival of the *mir-252*(*n4570*)*; mir-251*(*n4606*) was not significantly different from that in *mir-251*(*n4606*) (*P* = 0.9801) or *mir-252*(*n4570*) (*P* = 0.9703) mutant animals. (**b**) Genetic interaction between *mir-251* and *mir-252* in regulating SC5314 CFU in *C. albicans* infected nematodes. Live *C. albicans* SC5314 were recovered from *C. elegans* after 24 h of infection. (**c**) Genetic interaction between *mir-251* and *mir-252* in regulating *abf-2* expression in nematodes infected with *C. albicans* for 24 h. Bars represent means ± S.E.M. ^**^P < 0.01 *vs* N2.

In *C. elegans*, *mir-251* and *mir-252* belong to the same family. Finally, we investigated the genetic interaction between *mir-251* and *mir-252* in regulating the innate immune response in *C. albicans* infected nematodes. After *C. albicans* SC5314 infection, the lifespan in double mutant of *mir-252*(*n4570*)*; mir-251*(*n4606*) was similar to that in the single mutant of *mir-251*(*n4606*) or *mir-252*(*n4570*) (Fig. 8a). Likewise, the SC5314 CFU in *C. albicans* infected *mir-252*(*n4570*)*; mir-251*(*n4606*) was similar to that in *C. albicans* infected *mir-251*(*n4606*) or *mir-252*(*n4570*) (Fig. 8b). Moreover, the expression of *abf-2* in *C. albicans* infected *mir-252*(*n4570*)*; mir-251*(*n4606*) double mutant was also similar to that in *C. albicans* infected *mir-251*(*n4606*) or *mir-252*(*n4570*) mutant (Fig. 8c). Therefore, we did not observe the potential redundant function between *mir-251* and *mir-252* in regulating innate immunity in *C. albicans* infected nematodes. This may be at least partially due to the possibility that the *mir-252; mir-251* double mutant may have a worsened general fitness, which then prevents them from surviving better on *C. albicans*.

## Discussion

*C. elegans* has emerged as a powerful *in vivo* platform for the study of the innate immune response^5^,^6^. *C. elegans* can be infected with a wide variety of bacterial and fungal pathogens that interact with both the intestinal and epidermal epithelial cells^5^,^7^. This infection process leads to the activation of nematodes’ innate immune response, which involves the up-regulation of a series of proteins, including the proposed antimicrobial peptides such as the neuropeptide-like proteins (NLPs) and caenicin (CNC) family proteins^8^,^9^,^13^. *C. albicans* infection can reduce lifespan, and increase the expression of some antimicrobial genes^13^. In this study, we used the CaSA1 strain, which carries the *GFP* gene under the control of *CDR1* promoter^24^, to further examine the persistence of *C. albicans* cells within the pharynx and the intestine of *C. elegans*. In contrast to *E. coli* OP50 and heat-killed *C. albicans* CaSA1, live *C. albicans* CaSA1 cells accumulated dramatically in the pharyngeal grinder organ, and in the proximal, middle, and distal intestine of *C. elegans* (Fig. S1), which further implies that persistence of *C. albicans* cells in the pharynx and the intestine may be a critical step during *Candida* infection in *C. elegans*.

**Figure 9:**
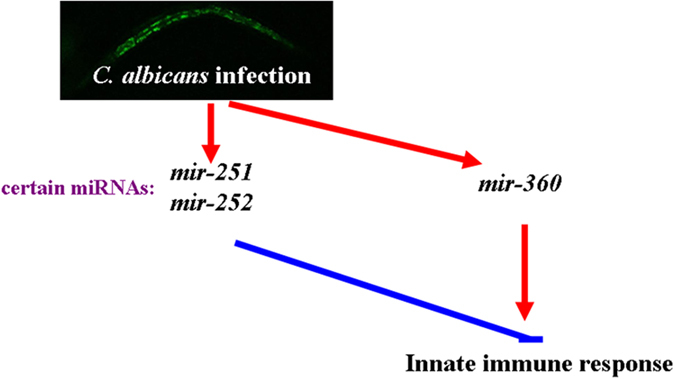
A diagram for the dysregulated miRNAs induced by *C. albicans* infection and their potential functions in regulating innate immunity in nematodes.

Some evidence indicates that *C. elegans* can mount a rapid innate immune response against pathogenic fungi^11^,^12^. Previous study has characterized the transcriptional response of *C. elegans* to *C. albicans* infection^13^. miRNAs are small RNAs that can regulate gene expression by inhibiting protein translation or by degrading mRNA transcripts^15^,^16^. We compared the miRNA expression profiles of nematodes infected with *C. albicans* SC5314 to control nematodes fed with a non-pathogenic food source, heat-killed *C. albicans* SC5314. We used a short (4 h) infection duration for miRNA profiling to maximize the yield of transcriptional changes associated with pathogen detection, rather than the transcriptional changes associated with intestinal damage^25^. Based on fold-change analysis and statistical analysis, we identified 16 up-regulated miRNAs (*mir-240*, *mir-75*, *mir-787*, *mir-62*, *mir-251*, *mir-252*, *mir-1821*, *mir-360*, *mir-353*, *mir-254*, *mir-229*, *mir-1824*, *mir-795*, *mir-1820*, *mir-41*, and *mir-4923b*) and 4 down-regulated miRNAs (*mir-4812*, *mir-53*, *mir-794*, and *mir-86*) in nematodes infected with *C. albicans* SC5314 (Figs 1, 2a,b and 9, Table S2). We confirmed the observed changes in some of these candidate miRNAs by qRT-PCR (Fig. 2c). A previous study has described the changes in mRNA profiles after nematodes were infected with *C. albicans* SC5314^13^. Together, these results will provide a strong basis to further elucidate how miRNA-mRNA networks control *C. elegans*’ innate immune response to *C. albicans* infection. In addition, the dysregulated miRNAs identified in this study will be further helpful for our understanding of the functions of the genes that are dysregulated by *C. albicans* infection in nematodes.

Our gene ontology and KEGG signaling analysis of the 20 miRNAs that were affected by *C. albicans* SC5314 infection implies that *C. albicans* infection can (at a minimum) affect the following biological processes: development, reproduction, intracellular organelle, cell cycle, cellular transportation, signal transduction, protein binding, cellular metabolism, cell communication, and response to stimulus (Fig. 3, Tables S3–S6). It is also possible that some important developmental and biochemical signaling pathways, such as the Wnt, Hedgehog, Notch, MAPK, mTOR, ErbB, Jak-STAT, TGF-beta, and calcium signaling pathways, and the mRNA surveillance pathway (Fig. 3b, Tables S3–S6), could play important roles in regulating the innate immune response to *C. albicans* infection in nematodes. Among these signaling pathways, the function of the p38 MAPK signaling pathway in regulating innate immune response to *C. albicans* has been confirmed^11^,^12^. Nevertheless, how most of these predicted signaling pathways regulate the innate immune response to *C. albicans* infection remains unclear. Moreover, our data imply that endocytosis, phagosomes, the proteasome, peroxisomes, and lysosomes play crucial roles in modulating the innate immune response to *C. albicans* infection in nematodes (Fig. 3b).

We also examined the adverse effects of *C. albicans* infection on lifespan in miRNA mutant nematodes. Among the available mutants for candidate miRNAs, we found that *mir-251* and *mir-252* mutants were resistant to *C. albicans* SC5314 infection, whereas *mir-360* mutants were hypersensitive to *C. albicans* SC5314 infection (Figs 4 and 9). These data suggest that mutations in *mir-251*, *mir-252*, or *mir-360* can influence the adverse effects of *C. albicans* infection on nematodes. Nevertheless, we cannot exclude the possibility that other miRNAs may also affect the pathogenesis of *C. albicans* infection in nematodes. In addition, due to the absence of available mutants, how some of the dysregulated miRNAs we identified affect the innate immune response remains unclear.

By examining a selection of antimicrobial genes (*abf-2*, *cnc-4*, *cnc-7*, and *fipr-22/33*), we examined the possible innate immune response of miRNA mutants to *C. albicans* SC5314 infection. After infection with *C. albicans* SC5314, *mir-251* and *mir-252* mutants exhibited higher expression levels of *abf-2, cnc-4*, *cnc-7*, and *fipr-22/23* compared to wild-type N2 (Figs 5a and 9). These results suggest that mutation of *mir-251* or *mir-252* can increase the innate immune response to *C. albicans* SC5314. In contrast, after infection with *C. albicans* SC5314, *mir-360* mutants showed lower expression levels of *abf-2, cnc-4*, *cnc-7*, and *fipr-22/23* than wild-type N2 (Fig. 5a), suggesting that mutation of *mir-360* may reduce the innate immune response to *C. albicans* SC5314. Meanwhile, after 4-h infection with *C. albicans* SC5314, we observed that mutation of *mir-360* did not obviously influence the expression level of any examined antimicrobial gene (data not shown), implying that we should consider both the early and late immune response of nematodes during *C. albicans* infection.

Using both the SC5314 and the CaSA1 strains, we further examined *C. albicans* colony formation in the intestine of candidate miRNA mutants. We determined the fungal burden in both the proximal and distal intestine in nematodes, because the egg formed would not influence our detection of *C. albicans* colonies in these regions of the intestine. Consistent with the effects of *mir-251*, *mir-252*, and *mir-360* mutations on lifespan and immune-response gene expression, mutation of *mir-251* and *mir-252* reduced *C. albicans* colony formation in the intestine, whereas mutation of *mir-360* enhanced *C. albicans* colony formation in the intestine (Fig. 6). Together, these results imply that the modulation of fungal burden may be closely associated with the changes in lifespan and innate immune-response gene expression in these miRNA mutants.

More interestingly, we found that *mir-251* and *mir-252* could function downstream of the p38 MAPK signaling pathway or the IGF-1/insulin-like signaling pathway to regulate innate immunity in nematodes after *C. albicans* infection (Fig. 9). The p38 MAPK signaling pathway has been shown to play a key role in regulating the innate immune response to *C. albicans* infection^11^,^12^. Previous studies have also demonstrated that the IGF-1/insulin-like signaling pathway is involved in the control of the innate immune response to pathogen infection in nematodes^8^,^10^,^11^. The identification of the relevant *mir-251* and *mir-252* targets will strengthen our understanding of the molecular mechanisms, such as the p38 MAPK and IGF-1/insulin-like signaling pathways, in regulating innate immunity in *C. albicans* infected nematodes. Furthermore, using the miRBase software (http://www.mirbase.org), we searched the potential targets for *mir-251* and *mir-252*. We found that DAF-16 may act as a direct target for *mir-251* and *mir-252*, which implies a feedback mechanism may exist between IGF-1/insulin-like signaling pathway and these two miRNAs in the control of innate immunity in nematodes.

In conclusion, using SOLiD sequencing, we profiled miRNA dysregulation after *C. albicans* infection in nematodes. Our bioinformatics analysis of gene ontology and KEGG signaling pathways implies that the dysregulated miRNAs may be involved in the control of some important biological processes including development, reproduction, intracellular organelle, cell cycle, cellular transportation, signal transduction, cellular metabolism, cell communication, and response to stimulus in nematodes infected with *C. albicans*. Using the available mutants, we found that *mir-251* or *mir-252* mutation renders nematodes resistant to *C. albicans* infection, whereas *mir-360* mutation makes nematodes hypersensitive to *C. albicans* infection. Our data suggest that *mir-251*, *mir-252*, and *mir-360* play crucial roles in regulating the innate immune response to *C. albicans* infection. In addition, *mir-251* and *mir-252* may function downstream of the p38 MAPK or IGF-1/insulin-like pathway to regulate innate immunity in *C. albicans-*infected nematodes. In *C. elegans*, *mir-251* and *mir-252* are the homologues of human miRNA of *mir-26*^26^. Therefore, our study provides an important molecular basis for further elucidating the miRNA-mRNA networks involved in the control of innate immunity of organisms in response to *C. albicans* infection.

## Methods

### Strains and media

*C. elegans* were maintained on nematode growth medium (NGM) plates seeded with *Escherichia coli* OP50 as described^27^. *C. elegans* strains used in this study were wild-type N2, *mir-251*(*n4606*), *mir-252*(*n4570*), *mir-254*(*n4470*), *mir-360*(*n4635*), *mir-240/786*(*n4541*), *mir-62*(*n4539*), and *mir-75*(*n4472*). The *C. albicans* strains used in this study were SC5314 (clinical isolate), a strain that is virulent towards *C. elegans*^28^, and *C. albicans* CaSA1 (ura3::imm434/ura3::imm434; *CDR1-GFP-URA3*). In *C. albicans*, the *CDR1* gene encodes an ABC transporter that functions as an efflux pump, which is involved in the control of pathogenic adaptation^24^. Unless otherwise specified, *C. albicans* SC5314 was used as the wild-type *C. albicans* strain. Yeast strains were grown in liquid yeast extract-peptone-dextrose (YPD) broth or on brain heart infusion (BHI) agar containing kanamycin (45 mg/mL) at 30 °C. Bacteria were grown in Luria Broth (LB).

### Small RNA extraction and SOLiD sequencing

A single colony of *C. albicans* SC5314 was used to inoculate 1 mL of YPD broth, which was allowed to grow overnight with agitation at 30 °C. Synchronized L1-larvae wild-type nematodes were plated on 10 cm NGM plates seeded with *E. coli* OP50 and grown at 20 °C until they were young adults. Nematodes were transferred onto plates containing 20 mL of BHI agar with kanamycin 45 (mg/mL) and either live *C. albicans* or heat-killed *C. albicans*. *C. albicans* cells (50 μL) were added together with 200 μL of PBS buffer to facilitate their even dispersion. Infection was performed for 4 h at 25 °C, and three replicates were performed.

After infection, nematodes were washed with sterile M9 buffer for five times, and then lysed to extract small RNA for an RNAomics assay. Small RNAs were extracted using mirVana^TM^ miRNA isolation kit (Ambion), and converted into a double-stranded cDNA library followed by an adaptor ligation. Whole-transcriptome libraries were constructed using TruSeq Stranded Total RNA with Rib-Zero Gold (Illumina, San Diego, CA, USA) according to the manufacturer’s instructions. Library quality was analyzed using an Agilent 2100 bioanalyzer after gel purification using Qiagen MinElute^®^ reaction cleanup kit and gel extraction kit before next-generation high-throughput sequencing with the Applied Biosystems SOLiD^TM^ system. SOLiD results developed by ABI were expressed as nucleotide sequences and their coverage. First, the 50 nt sequence tags from Illumina sequencing underwent data cleaning, which excludes poor quality reads, 3′ adaptor reads, reads with 5′ adaptor contaminants, and reads shorter than 18 nt. The remaining sequences were mapped to the *Caenorhabditis elegans* genome using the SOAP program (http://soap.genomics.org.cn) with a tolerance of one mismatch. The matched sequences were blasted against the Rfam and NCBI GenBank databases to filter out rRNAs, tRNAs, snRNAs and snoRNAs. The sequences originating from repetitive genomic elements were also filtered by RepeatMasker software. The remaining reads were aligned to *C. elegans* miRNA precursor sequences in the miRBase (v21) database and read counts were calculated for each miRNA.

### Bioinformatics analysis

We compared miRNA expression in nematodes exposed to live *C. albicans* yeast with control nematodes fed heat-killed *C. albicans*. Changes in miRNA expression after live *C. albicans* feeding were analyzed by DESeq (an R package to estimate the variance and to test for differential expression). We identified microRNAs that were up- or down-regulated using a 2-fold, statistically significant (*P* < 0.01) change as our cutoff. The data were then plotted as a scatter diagram after normalization. The predicted targeted genes of miRNAs that changed after *C. albicans* infection were analyzed using the TargetScan database (http://www.targetscan.org). The predicted targeted genes prominently affected were classified in terms of their gene ontology biological processes and the KEGG signaling pathways using the corresponding bioinformatics tools (http://www.geneontology.org and http://www.genome.jp/kegg/), using a statistical significance cut-off of *P* < 0.01.

### *C. elegans* survival assay

*C. elegans* survival analysis was performed as previously described^13^,^29^. *C. albicans* was seeded on plates containing brain heart infusion (BHI) and kanamycin (45 μg/mL). Age-synchronous populations of young adults were washed from NGM plates containing their food source (*E. coli.* OP50) with M9 buffer, and added to the center of the *C. albicans* lawns. In the assay plates, 75 μg/mL of fluoro-29-deoxyuridine (FUdR) was added to prevent the growth of progeny. Infection was performed for 4 h at 25 °C after adding 60 animals to the plate. Next, animals were transferred into a single well of a tissue culture plate (Corning, Inc) containing 2 mL of liquid medium (80% M9, and 20% BHI) and kanamycin (45 μg/mL). Nematodes were scored as dead or live every 24 h. Nematodes would be scored as dead if no response was detected after prodding with a platinum wire. Three replicates were analyzed for each experiment.

### *C. albicans* colony formation assay

The number of *C. albicans* CFU in *C. elegans* was quantified based on the protocol described previously^30^. Young adults were infected with *C. albicans* lawns for 24 h. After washing with sterile M9 buffer for five times to remove surface *C. albicans*, each group of 50 nematodes was homogenized using a homogenizer and plated on a YPD agar containing kanamycin (45 μg/mL), ampicillin (100 μg/mL), and streptomycin (100 μg/mL). The plates were incubated for 48 h at 37 °C. *C. albicans* colonies were counted to determine the CFU per nematode. Ten replicates were analyzed for each experiment.

### Microscopic assay of *C. elegans*

After preparation of a 2% agarose pad containing 0.01 M sodium azide on a slide, 5 μL M9 buffer was added to the pad. Animals infected with *C. albicans* were picked and transferred to the M9 drop on the pad. The mounted animals were covered with a coverslip and observed using an Axiovision Zeiss microscope under differential interference contrast and epifluorescence optics.

### Reverse transcription and qRT-PCR

Total RNA was extracted from nematodes according to the manufacturer’s protocol in RNeasy Mini Kit (Qiagen). Purity and concentration of RNAs were analyzed by OD 260/280 in a spectrophotometer. cDNA was synthesized in a 12.5 μL reaction volume containing 625 ng total RNA, 0.5 mM reverse-transcript primers, 50 mM Tris-HCl, 75 mM KCl, 3 mM MgCl_2_, 10 mM dithiothreitol, 20 units of ribonuclease inhibitor, and 100 units of reverse transcriptase (Takara, China). The reaction mixture was first incubated at 25 °C for 5 min and 42 °C for 60 min. Again, the reverse transcriptase was inactivated at 70 °C for 15 min. Transcriptional quantification was determined by real-time PCR in an ABI 7500 real-time PCR system using Evagreen (Biotium, USA). Primer information for miRNAs was shown in Tables S7 and 8. The miRNA expressions were expressed as the relative expression ratio between certain miRNA and *F35C11.9* encoding a small nuclear RNA U6. The related information for antimicrobial genes was shown in Table S9. The final results for antimicrobial genes were expressed as relative expression ratio between targeted genes and reference *act-1* gene.

### Statistical analysis

All data in the present study were presented as means ± standard error of the mean (SEM). Graphs were prepared with Microsoft Excel software (Microsoft Corp., Redmond, WA). Statistical analysis was performed with aid of SPSS 12.0 software (SPSS Inc., Chicage, USA). Differences between groups were determined using analysis of variance (ANOVA), and probability levels of 0.05 and 0.01 were considered statistically significant. Lifespan data were statistically analyzed using 2-tailed 2 sample t-test assay (Minitab Ltd., Coventry, UK).

## Additional Information

**How to cite this article**: Sun, L. *et al*. microRNAs Involved in the Control of Innate Immunity in *Candida* Infected *Caenorhabditis elegans*. *Sci. Rep.* **6**, 36036; doi: 10.1038/srep36036 (2016).

**Publisher’s note:** Springer Nature remains neutral with regard to jurisdictional claims in published maps and institutional affiliations.

## Supplementary Material

Supplementary Information
