# Supplementary material for: microRNAs Involved in the Control of Innate Immunity in Candida Infected Caenorhabditis elegans
Source: Sci Rep. 2016 Oct 31;6:36036. doi: 10.1038/srep36036 (PMC5086856; doi:10.1038/srep36036)
Supplement: Supplementary Information [file srep36036-s1.doc]

**microRNAs Involved in the Control of Innate Immunity in *Candida* Infected *Caenorhabditis elegans***

Lingmei Sun,^*^ Lingtong Zhi,^*^ Shumaila Shakoor,^*^ Kai Liao & Dayong Wang^**^

Key Laboratory of Developmental Genes and Human Disease in Ministry of Education, Medical School, Southeast University, Nanjing 210009, China

^*^They contributed equally to this work.

^**^Correspondence: D-Y Wang, Medical School, Southeast University, Nanjing 210009, China. E-mail: [dayongw@seu.edu.cn](mailto:dayongw@seu.edu.cn)

**Supporting Information:**


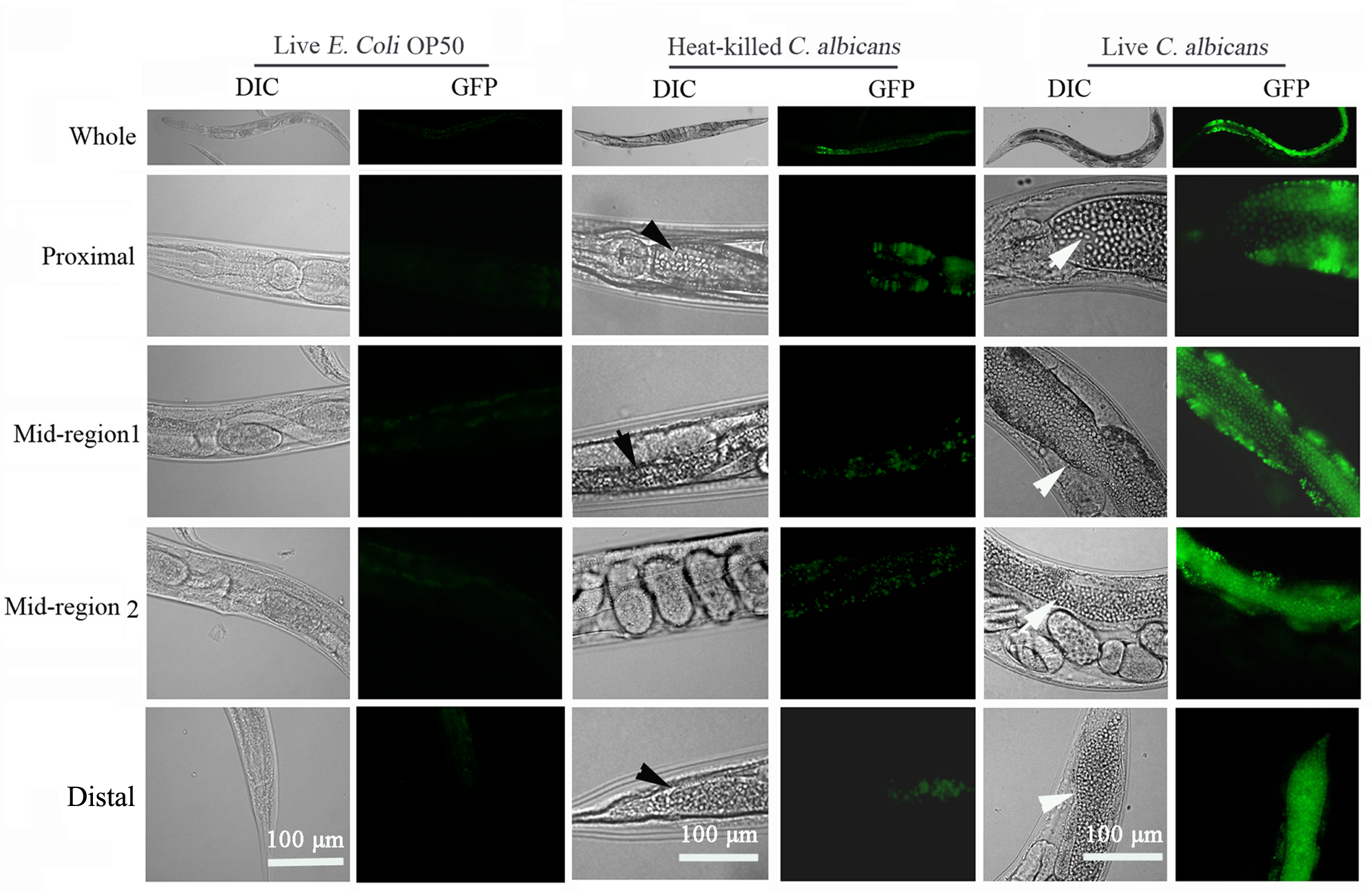


**Figure S1. Intact *C. albicans* cells in the body of *C. elegans*.**  Young adults of wild-type *C. elegans* were fed with *E. coli* OP50, heat-killed *C. albicans* CaSA1, or live *C. albicans* CaSA1 for 16 h. Arrows indicate the intact *C. albicans* cells in the intestine of *C. elegans*.


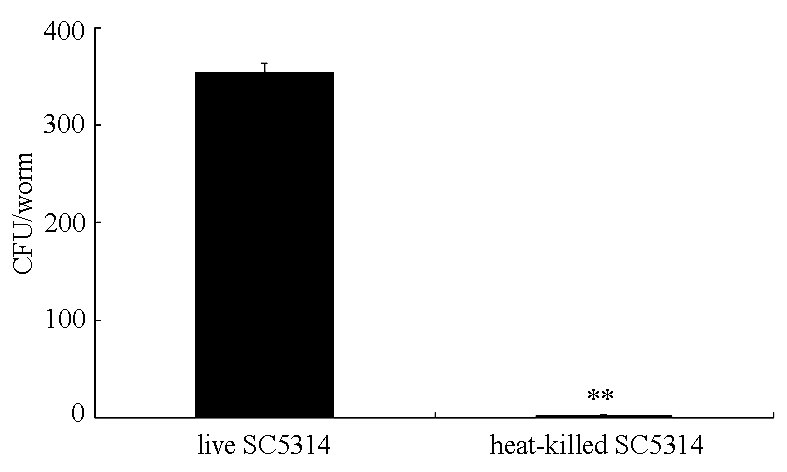


**Figure S2. Comparison of CFU between live *C. albicans* SC5314 and heat-killed *C. albicans* SC5314 in wild-type N2 nematodes.**  Live or heat-killed *C. albicans* SC5314 was recovered from *C. elegans* after 24 h of infection. Bars represent means ± S.E.M. ^**^*P* < 0.01 *vs* live SC5314.


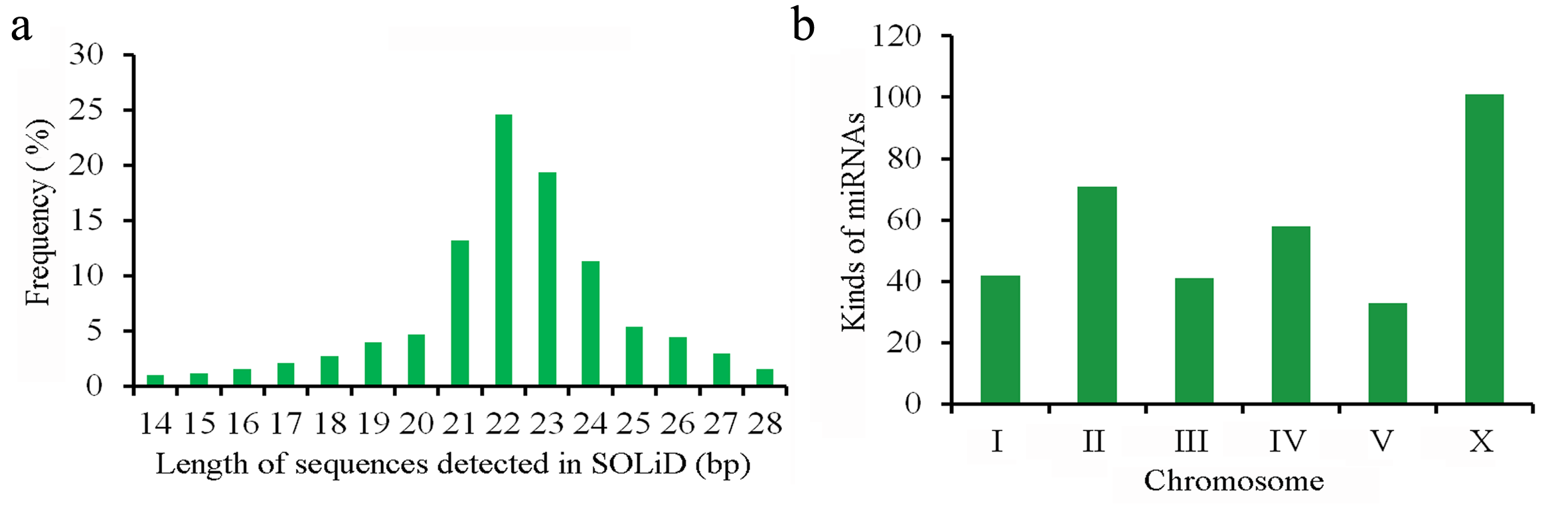


**Figure S3. Length distribution (a) and chromosome localization (b) of miRNAs detected by the SOLiD sequencing.**


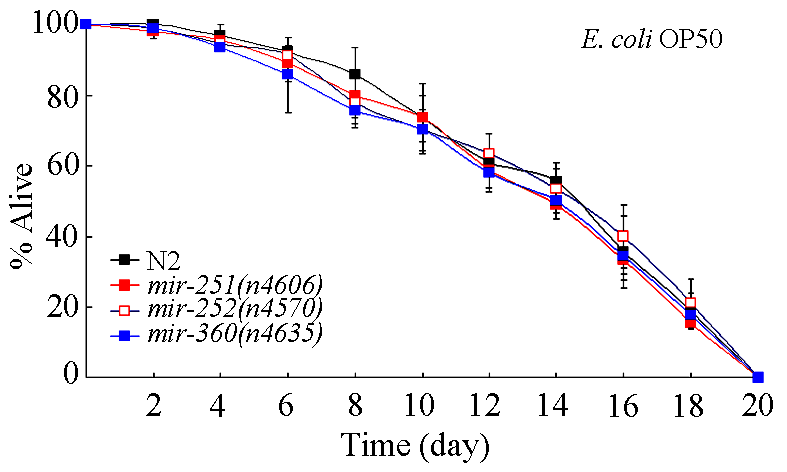


**Figure S4. Lifespan of wild-type, *mir-251*, *mir-252*, and *mir-360* mutants in plates fed with *E. coli* OP50.**  Bars represent means ± S.E.M.

**Table S1. miRNAs identified with their read counts.**

| NO. | Accession | Heat-killed *C. albicans* | Live *C. albicans* |
| --- | --- | --- | --- |
| 1 | cel-let-7-3p | 323 | 179 |
| 2 | cel-let-7-5p | 25442 | 15397 |
| 3 | cel-lin-4-3p | 60 | 18 |
| 4 | cel-lin-4-5p | 55887 | 25998 |
| 5 | cel-lsy-6 | 17 | 17 |
| 6 | cel-miR-1018 | 100 | 96 |
| 7 | cel-miR-1019-3p | 0 | 0 |
| 8 | cel-miR-1019-5p | 0 | 0 |
| 9 | cel-miR-1020-3p | 225 | 120 |
| 10 | cel-miR-1020-5p | 69 | 40 |
| 11 | cel-miR-1021 | 0 | 0 |
| 12 | cel-miR-1022-3p | 0 | 0 |
| 13 | cel-miR-1022-5p | 880 | 808 |
| 14 | cel-miR-124-3p | 958 | 646 |
| 15 | cel-miR-124-5p | 16 | 6 |
| 16 | cel-miR-1-3p | 828246 | 463719 |
| 17 | cel-miR-1-5p | 3289 | 2091 |
| 18 | cel-miR-1817 | 396 | 364 |
| 19 | cel-miR-1818 | 0 | 1 |
| 20 | cel-miR-1819-3p | 213 | 125 |
| 21 | cel-miR-1819-5p | 7 | 8 |
| 22 | cel-miR-1820-3p | 16 | 4 |
| 23 | cel-miR-1820-5p | 605 | 1389 |
| 24 | cel-miR-1821 | 66 | 117 |
| 25 | cel-miR-1822-3p | 474 | 218 |
| 26 | cel-miR-1822-5p | 14 | 21 |
| 27 | cel-miR-1823 | 4 | 0 |
| 28 | cel-miR-1824-3p | 8 | 0 |
| 29 | cel-miR-1824-5p | 37 | 78 |
| 30 | cel-miR-1828 | 1 | 0 |
| 31 | cel-miR-1829a-3p | 37 | 16 |
| 32 | cel-miR-1829a-5p | 135 | 105 |
| 33 | cel-miR-1829b | 443 | 413 |
| 34 | cel-miR-1829c | 668 | 411 |
| 35 | cel-miR-1830-3p | 797 | 478 |
| 36 | cel-miR-1830-5p | 27 | 30 |
| 37 | cel-miR-1832a | 0 | 0 |
| 38 | cel-miR-1832b-3p | 0 | 0 |
| 39 | cel-miR-1832b-5p | 0 | 0 |
| 40 | cel-miR-1833 | 0 | 0 |
| 41 | cel-miR-2207-3p | 0 | 0 |
| 42 | cel-miR-2207-5p | 0 | 0 |
| 43 | cel-miR-2208a-3p | 0 | 0 |
| 44 | cel-miR-2208a-5p | 0 | 0 |
| 45 | cel-miR-2208b-3p | 0 | 0 |
| 46 | cel-miR-2208b-5p | 0 | 0 |
| 47 | cel-miR-2209a-3p | 6 | 3 |
| 48 | cel-miR-2209a-5p | 0 | 0 |
| 49 | cel-miR-2209b-3p | 0 | 0 |
| 50 | cel-miR-2209b-5p | 0 | 0 |
| 51 | cel-miR-2209c-3p | 8 | 0 |
| 52 | cel-miR-2209c-5p | 0 | 0 |
| 53 | cel-miR-2210-3p | 15 | 0 |
| 54 | cel-miR-2210-5p | 0 | 0 |
| 55 | cel-miR-2211-3p | 0 | 0 |
| 56 | cel-miR-2211-5p | 0 | 0 |
| 57 | cel-miR-2212-3p | 0 | 0 |
| 58 | cel-miR-2212-5p | 179 | 116 |
| 59 | cel-miR-2213-3p | 0 | 0 |
| 60 | cel-miR-2213-5p | 8 | 8 |
| 61 | cel-miR-2214-3p | 5 | 5 |
| 62 | cel-miR-2214-5p | 37 | 50 |
| 63 | cel-miR-2215-3p | 0 | 0 |
| 64 | cel-miR-2215-5p | 0 | 0 |
| 65 | cel-miR-2216-3p | 0 | 0 |
| 66 | cel-miR-2216-5p | 18 | 1 |
| 67 | cel-miR-2217-3p | 26 | 6 |
| 68 | cel-miR-2217-5p | 0 | 0 |
| 69 | cel-miR-2218a-3p | 0 | 0 |
| 70 | cel-miR-2218a-5p | 0 | 0 |
| 71 | cel-miR-2218b-3p | 3 | 0 |
| 72 | cel-miR-2218b-5p | 0 | 0 |
| 73 | cel-miR-2219-3p | 0 | 5 |
| 74 | cel-miR-2219-5p | 0 | 1 |
| 75 | cel-miR-2220-3p | 24 | 21 |
| 76 | cel-miR-2220-5p | 0 | 0 |
| 77 | cel-miR-2221 | 7 | 0 |
| 78 | cel-miR-228-3p | 63 | 32 |
| 79 | cel-miR-228-5p | 167417 | 66680 |
| 80 | cel-miR-229-3p | 591 | 283 |
| 81 | cel-miR-229-5p | 7961 | 16048 |
| 82 | cel-miR-230-3p | 3138 | 2114 |
| 83 | cel-miR-230-5p | 3574 | 1502 |
| 84 | cel-miR-231-3p | 811 | 514 |
| 85 | cel-miR-231-5p | 133 | 48 |
| 86 | cel-miR-232-3p | 2323 | 2391 |
| 87 | cel-miR-232-5p | 7 | 6 |
| 88 | cel-miR-233-3p | 4569 | 2501 |
| 89 | cel-miR-233-5p | 11 | 11 |
| 90 | cel-miR-234-3p | 2091 | 1256 |
| 91 | cel-miR-234-5p | 1 | 0 |
| 92 | cel-miR-235-3p | 50976 | 26041 |
| 93 | cel-miR-235-5p | 1 | 0 |
| 94 | cel-miR-236-3p | 3668 | 3089 |
| 95 | cel-miR-236-5p | 14 | 35 |
| 96 | cel-miR-237-3p | 120 | 55 |
| 97 | cel-miR-237-5p | 171865 | 109806 |
| 98 | cel-miR-238-3p | 32624 | 21139 |
| 99 | cel-miR-238-5p | 161 | 55 |
| 100 | cel-miR-239a-3p | 66 | 67 |
| 101 | cel-miR-239a-5p | 17107 | 16670 |
| 102 | cel-miR-239b-3p | 5 | 9 |
| 103 | cel-miR-239b-5p | 901 | 476 |
| 104 | cel-miR-2-3p | 83217 | 102315 |
| 105 | cel-miR-240-3p | 3143 | 4479 |
| 106 | cel-miR-240-5p | 914 | 557 |
| 107 | cel-miR-241-3p | 1442 | 1436 |
| 108 | cel-miR-241-5p | 6031 | 2883 |
| 109 | cel-miR-242 | 1393 | 1014 |
| 110 | cel-miR-243-3p | 856 | 516 |
| 111 | cel-miR-243-5p | 1499 | 499 |
| 112 | cel-miR-244-3p | 127 | 59 |
| 113 | cel-miR-244-5p | 7455 | 5826 |
| 114 | cel-miR-245-3p | 669 | 674 |
| 115 | cel-miR-245-5p | 0 | 0 |
| 116 | cel-miR-246-3p | 5929 | 2861 |
| 117 | cel-miR-246-5p | 8 | 9 |
| 118 | cel-miR-247-3p | 12136 | 10775 |
| 119 | cel-miR-247-5p | 13 | 34 |
| 120 | cel-miR-248 | 996 | 729 |
| 121 | cel-miR-249-3p | 97 | 96 |
| 122 | cel-miR-249-5p | 0 | 1 |
| 123 | cel-miR-250-3p | 16383 | 6904 |
| 124 | cel-miR-250-5p | 63 | 41 |
| 125 | cel-miR-251 | 154 | 249 |
| 126 | cel-miR-252-3p | 0 | 0 |
| 127 | cel-miR-252-5p | 263 | 428 |
| 128 | cel-miR-253-3p | 1361 | 579 |
| 129 | cel-miR-253-5p | 0 | 0 |
| 130 | cel-miR-254 | 672 | 1352 |
| 131 | cel-miR-255-3p | 72 | 63 |
| 132 | cel-miR-255-5p | 0 | 2 |
| 133 | cel-miR-256 | 0 | 0 |
| 134 | cel-miR-257 | 0 | 0 |
| 135 | cel-miR-258 | 0 | 0 |
| 136 | cel-miR-259-3p | 16 | 34 |
| 137 | cel-miR-259-5p | 989 | 775 |
| 138 | cel-miR-2-5p | 93 | 27 |
| 139 | cel-miR-260 | 7 | 16 |
| 140 | cel-miR-261 | 0 | 0 |
| 141 | cel-miR-262 | 3 | 0 |
| 142 | cel-miR-264 | 0 | 0 |
| 143 | cel-miR-265 | 0 | 0 |
| 144 | cel-miR-266 | 0 | 0 |
| 145 | cel-miR-267 | 0 | 0 |
| 146 | cel-miR-268 | 0 | 0 |
| 147 | cel-miR-269 | 0 | 0 |
| 148 | cel-miR-270 | 0 | 0 |
| 149 | cel-miR-271 | 0 | 0 |
| 150 | cel-miR-272 | 0 | 0 |
| 151 | cel-miR-273 | 0 | 0 |
| 152 | cel-miR-2953-3p | 1 | 0 |
| 153 | cel-miR-2953-5p | 12 | 5 |
| 154 | cel-miR-34-3p | 4778 | 1702 |
| 155 | cel-miR-34-5p | 3665 | 2784 |
| 156 | cel-miR-353 | 40 | 79 |
| 157 | cel-miR-35-3p | 1132 | 740 |
| 158 | cel-miR-354 | 8 | 4 |
| 159 | cel-miR-355 | 5248 | 2562 |
| 160 | cel-miR-35-5p | 40 | 23 |
| 161 | cel-miR-356a | 0 | 0 |
| 162 | cel-miR-356b-3p | 87 | 108 |
| 163 | cel-miR-356b-5p | 6 | 0 |
| 164 | cel-miR-357-3p | 153 | 69 |
| 165 | cel-miR-357-5p | 0 | 0 |
| 166 | cel-miR-358-3p | 244 | 101 |
| 167 | cel-miR-358-5p | 87 | 61 |
| 168 | cel-miR-359 | 291 | 119 |
| 169 | cel-miR-360-3p | 0 | 9 |
| 170 | cel-miR-360-5p | 50 | 96 |
| 171 | cel-miR-36-3p | 551 | 397 |
| 172 | cel-miR-36-5p | 16 | 21 |
| 173 | cel-miR-37-3p | 1992 | 887 |
| 174 | cel-miR-37-5p | 10 | 3 |
| 175 | cel-miR-38-3p | 341 | 192 |
| 176 | cel-miR-38-5p | 0 | 7 |
| 177 | cel-miR-392-3p | 8 | 14 |
| 178 | cel-miR-392-5p | 0 | 0 |
| 179 | cel-miR-39-3p | 343 | 159 |
| 180 | cel-miR-39-5p | 33 | 9 |
| 181 | cel-miR-40-3p | 358 | 226 |
| 182 | cel-miR-40-5p | 3 | 1 |
| 183 | cel-miR-41-3p | 47 | 29 |
| 184 | cel-miR-41-5p | 3 | 17 |
| 185 | cel-miR-42-3p | 9442 | 5162 |
| 186 | cel-miR-42-5p | 1 | 1 |
| 187 | cel-miR-43-3p | 1711 | 1812 |
| 188 | cel-miR-43-5p | 3 | 2 |
| 189 | cel-miR-44-3p | 314649 | 206406 |
| 190 | cel-miR-44-5p | 146 | 86 |
| 191 | cel-miR-45-3p | 314688 | 206472 |
| 192 | cel-miR-45-5p | 83 | 32 |
| 193 | cel-miR-46-3p | 20995 | 14857 |
| 194 | cel-miR-46-5p | 2285 | 1037 |
| 195 | cel-miR-47-3p | 16622 | 11144 |
| 196 | cel-miR-47-5p | 1759 | 932 |
| 197 | cel-miR-4805-3p | 1 | 0 |
| 198 | cel-miR-4805-5p | 0 | 0 |
| 199 | cel-miR-4806-3p | 48 | 32 |
| 200 | cel-miR-4806-5p | 0 | 0 |
| 201 | cel-miR-4807 | 0 | 0 |
| 202 | cel-miR-4808-3p | 2 | 0 |
| 203 | cel-miR-4808-5p | 0 | 0 |
| 204 | cel-miR-4809-3p | 4 | 0 |
| 205 | cel-miR-4809-5p | 0 | 0 |
| 206 | cel-miR-4810 | 0 | 0 |
| 207 | cel-miR-4811-3p | 0 | 0 |
| 208 | cel-miR-4811-5p | 0 | 0 |
| 209 | cel-miR-4812-3p | 0 | 0 |
| 210 | cel-miR-4812-5p | 20 | 2 |
| 211 | cel-miR-4813-3p | 274 | 122 |
| 212 | cel-miR-4813-5p | 22 | 4 |
| 213 | cel-miR-4814-3p | 2 | 0 |
| 214 | cel-miR-4814-5p | 7 | 15 |
| 215 | cel-miR-4815 | 0 | 0 |
| 216 | cel-miR-4816-3p | 48 | 22 |
| 217 | cel-miR-4816-5p | 26 | 4 |
| 218 | cel-miR-48-3p | 557 | 477 |
| 219 | cel-miR-48-5p | 169571 | 99277 |
| 220 | cel-miR-4920 | 13 | 30 |
| 221 | cel-miR-4921 | 0 | 0 |
| 222 | cel-miR-4922 | 6 | 0 |
| 223 | cel-miR-4923a | 0 | 0 |
| 224 | cel-miR-4923b | 2 | 14 |
| 225 | cel-miR-4924 | 0 | 5 |
| 226 | cel-miR-4925 | 0 | 0 |
| 227 | cel-miR-4926 | 45 | 7 |
| 228 | cel-miR-4927 | 0 | 3 |
| 229 | cel-miR-4929 | 55 | 12 |
| 230 | cel-miR-4930 | 0 | 0 |
| 231 | cel-miR-4931 | 0 | 0 |
| 232 | cel-miR-4932 | 0 | 0 |
| 233 | cel-miR-4933 | 0 | 0 |
| 234 | cel-miR-4934 | 0 | 0 |
| 235 | cel-miR-4935 | 0 | 0 |
| 236 | cel-miR-4936 | 0 | 0 |
| 237 | cel-miR-4937 | 45 | 17 |
| 238 | cel-miR-4938 | 0 | 0 |
| 239 | cel-miR-49-3p | 8805 | 5129 |
| 240 | cel-miR-49-5p | 33 | 28 |
| 241 | cel-miR-50-3p | 9 | 0 |
| 242 | cel-miR-50-5p | 13547 | 8783 |
| 243 | cel-miR-51-3p | 111 | 57 |
| 244 | cel-miR-51-5p | 41267 | 32279 |
| 245 | cel-miR-52-3p | 392 | 116 |
| 246 | cel-miR-52-5p | 290245 | 286636 |
| 247 | cel-miR-53-3p | 299 | 62 |
| 248 | cel-miR-53-5p | 20016 | 18896 |
| 249 | cel-miR-54-3p | 295377 | 242814 |
| 250 | cel-miR-54-5p | 2976 | 1088 |
| 251 | cel-miR-55-3p | 15088 | 11107 |
| 252 | cel-miR-5545-3p | 0 | 5 |
| 253 | cel-miR-5545-5p | 1 | 3 |
| 254 | cel-miR-5546-3p | 0 | 0 |
| 255 | cel-miR-5546-5p | 0 | 0 |
| 256 | cel-miR-5547-3p | 32 | 17 |
| 257 | cel-miR-5547-5p | 11 | 7 |
| 258 | cel-miR-5548-3p | 0 | 0 |
| 259 | cel-miR-5548-5p | 13 | 0 |
| 260 | cel-miR-5549-3p | 0 | 0 |
| 261 | cel-miR-5549-5p | 0 | 0 |
| 262 | cel-miR-5550-3p | 0 | 0 |
| 263 | cel-miR-5550-5p | 0 | 0 |
| 264 | cel-miR-5551-3p | 0 | 0 |
| 265 | cel-miR-5551-5p | 256 | 115 |
| 266 | cel-miR-5552-3p | 0 | 0 |
| 267 | cel-miR-5552-5p | 0 | 0 |
| 268 | cel-miR-5553-3p | 0 | 0 |
| 269 | cel-miR-5553-5p | 2 | 4 |
| 270 | cel-miR-55-5p | 139 | 41 |
| 271 | cel-miR-5592-3p | 1301 | 788 |
| 272 | cel-miR-5592-5p | 412 | 220 |
| 273 | cel-miR-5593-3p | 0 | 0 |
| 274 | cel-miR-5593-5p | 0 | 0 |
| 275 | cel-miR-5594-3p | 17 | 3 |
| 276 | cel-miR-5594-5p | 22 | 3 |
| 277 | cel-miR-5595-3p | 0 | 0 |
| 278 | cel-miR-5595-5p | 0 | 0 |
| 279 | cel-miR-56-3p | 17425 | 10342 |
| 280 | cel-miR-56-5p | 218 | 186 |
| 281 | cel-miR-57-3p | 23 | 5 |
| 282 | cel-miR-57-5p | 62110 | 53695 |
| 283 | cel-miR-58-3p | 2610326 | 1525782 |
| 284 | cel-miR-58-5p | 1143 | 725 |
| 285 | cel-miR-58b-3p | 29 | 25 |
| 286 | cel-miR-58b-5p | 0 | 0 |
| 287 | cel-miR-59-3p | 15778 | 10637 |
| 288 | cel-miR-59-5p | 57 | 24 |
| 289 | cel-miR-60-3p | 182586 | 193462 |
| 290 | cel-miR-60-5p | 55 | 19 |
| 291 | cel-miR-61-3p | 34077 | 18821 |
| 292 | cel-miR-61-5p | 48 | 47 |
| 293 | cel-miR-62 | 2270 | 3448 |
| 294 | cel-miR-63-3p | 3757 | 2709 |
| 295 | cel-miR-63-5p | 2368 | 2092 |
| 296 | cel-miR-64-3p | 64 | 29 |
| 297 | cel-miR-64-5p | 41234 | 14524 |
| 298 | cel-miR-65-3p | 522 | 496 |
| 299 | cel-miR-65-5p | 80197 | 27947 |
| 300 | cel-miR-66-3p | 295 | 150 |
| 301 | cel-miR-66-5p | 33565 | 28028 |
| 302 | cel-miR-67-3p | 5993 | 6021 |
| 303 | cel-miR-67-5p | 10 | 28 |
| 304 | cel-miR-70-3p | 10005 | 6555 |
| 305 | cel-miR-70-5p | 13 | 3 |
| 306 | cel-miR-71-3p | 1336 | 1457 |
| 307 | cel-miR-71-5p | 117344 | 85250 |
| 308 | cel-miR-72-3p | 100 | 70 |
| 309 | cel-miR-72-5p | 73219 | 35806 |
| 310 | cel-miR-73-3p | 6664 | 4976 |
| 311 | cel-miR-73-5p | 35 | 14 |
| 312 | cel-miR-74-3p | 5501 | 5330 |
| 313 | cel-miR-74-5p | 9 | 23 |
| 314 | cel-miR-75-3p | 3186 | 4567 |
| 315 | cel-miR-75-5p | 153 | 72 |
| 316 | cel-miR-76-3p | 527 | 543 |
| 317 | cel-miR-76-5p | 1 | 1 |
| 318 | cel-miR-77-3p | 35179 | 21404 |
| 319 | cel-miR-77-5p | 118 | 27 |
| 320 | cel-miR-78 | 15 | 1 |
| 321 | cel-miR-784-3p | 0 | 0 |
| 322 | cel-miR-784-5p | 606 | 430 |
| 323 | cel-miR-785 | 1282 | 1054 |
| 324 | cel-miR-786-3p | 110 | 89 |
| 325 | cel-miR-786-5p | 2 | 0 |
| 326 | cel-miR-787-3p | 729 | 1069 |
| 327 | cel-miR-787-5p | 0 | 2 |
| 328 | cel-miR-788-3p | 293 | 294 |
| 329 | cel-miR-788-5p | 1502 | 1511 |
| 330 | cel-miR-789 | 112 | 30 |
| 331 | cel-miR-790-3p | 72 | 20 |
| 332 | cel-miR-790-5p | 444 | 307 |
| 333 | cel-miR-791-3p | 93 | 85 |
| 334 | cel-miR-791-5p | 0 | 1 |
| 335 | cel-miR-792-3p | 272 | 289 |
| 336 | cel-miR-792-5p | 0 | 0 |
| 337 | cel-miR-793 | 1348 | 1376 |
| 338 | cel-miR-79-3p | 25713 | 25060 |
| 339 | cel-miR-794-3p | 2 | 9 |
| 340 | cel-miR-794-5p | 292 | 70 |
| 341 | cel-miR-795-3p | 20 | 17 |
| 342 | cel-miR-795-5p | 32 | 69 |
| 343 | cel-miR-79-5p | 244 | 129 |
| 344 | cel-miR-796 | 0 | 0 |
| 345 | cel-miR-797-3p | 88 | 66 |
| 346 | cel-miR-797-5p | 1265 | 1093 |
| 347 | cel-miR-798 | 25 | 17 |
| 348 | cel-miR-799 | 1 | 0 |
| 349 | cel-miR-800-3p | 3 | 9 |
| 350 | cel-miR-800-5p | 2 | 1 |
| 351 | cel-miR-80-3p | 753633 | 611162 |
| 352 | cel-miR-80-5p | 3486 | 1798 |
| 353 | cel-miR-81-3p | 78299 | 49956 |
| 354 | cel-miR-81-5p | 304 | 102 |
| 355 | cel-miR-82-3p | 161405 | 88767 |
| 356 | cel-miR-82-5p | 83 | 40 |
| 357 | cel-miR-83-3p | 4489 | 3370 |
| 358 | cel-miR-83-5p | 24 | 14 |
| 359 | cel-miR-84-3p | 154 | 108 |
| 360 | cel-miR-84-5p | 9257 | 11150 |
| 361 | cel-miR-85-3p | 27136 | 30951 |
| 362 | cel-miR-85-5p | 38 | 22 |
| 363 | cel-miR-86-3p | 489 | 129 |
| 364 | cel-miR-86-5p | 24591 | 18017 |
| 365 | cel-miR-87-3p | 2821 | 1789 |
| 366 | cel-miR-87-5p | 3 | 7 |
| 367 | cel-miR-90-3p | 37835 | 26191 |
| 368 | cel-miR-90-5p | 17 | 32 |

**Table S2. The dysregulated miRNAs induce by *C. albicans* infection**

| Accession | Fold changes (FC) | Regulation | *P* value |
| --- | --- | --- | --- |
| *mir-4923b* | 5.2812 | up | 0.0041492583 |
| *mir-41* | 4.9125 | up | 0.005442629 |
| *mir-1820* | 3.4995 | up | 0.004234868 |
| *mir-795* | 3.2867 | up | 0.008474828 |
| *mir-1824* | 3.2133 | up | 0.0017698613 |
| *mir-229* | 3.0726 | up | 0.003040118 |
| *mir-254* | 3.0666 | up | 0.0010078422 |
| *mir-353* | 3.0104 | up | 0.009107861 |
| *mir-360* | 2.9265 | up | 0.0017898518 |
| *mir-1821* | 2.7021 | up | 0.0018285953 |
| *mir-252* | 2.4805 | up | 0.005183157 |
| *mir-251* | 2.4645 | up | 0.006695662 |
| *mir-62* | 2.3152 | up | 0.0040156213 |
| *mir-787* | 2.2351 | up | 0.0016476715 |
| *mir-75* | 2.1849 | up | 0.0041231526 |
| *mir-240* | 2.1721 | up | 0.0050195225 |
| *mir-86* | -2.5742 | down | 0.0015819919 |
| *mir-794* | -2.7367 | down | 0.005041952 |
| *mir-53* | -3.3638 | down | 0.0027789573 |
| *mir-4812* | -9.8795 | down | 0.0018172515 |

**Table S3. Gene ontology terms with gene counts more than 10 based on down-regulated miRNAs induced by *C. albicans* infection**

| No. | GO accession | GO term | Count |
| --- | --- | --- | --- |
| 1 | GO:0044699 | single-organism process | 107 |
| 2 | GO:0044763 | single-organism cellular process | 89 |
| 3 | GO:0065007 | biological regulation | 68 |
| 4 | GO:0050789 | regulation of biological process | 66 |
| 5 | GO:0050794 | regulation of cellular process | 60 |
| 6 | GO:0044260 | cellular macromolecule metabolic process | 53 |
| 7 | GO:0032501 | multicellular organismal process | 51 |
| 8 | GO:0044707 | single-multicellular organism process | 49 |
| 9 | GO:0044767 | single-organism developmental process | 47 |
| 10 | GO:0032502 | developmental process | 47 |
| 11 | GO:0007275 | multicellular organismal development | 44 |
| 12 | GO:0051179 | localization | 40 |
| 13 | GO:1901360 | organic cyclic compound metabolic process | 38 |
| 14 | GO:0046483 | heterocycle metabolic process | 37 |
| 15 | GO:0019222 | regulation of metabolic process | 36 |
| 16 | GO:0006139 | nucleobase-containing compound metabolic process | 36 |
| 17 | GO:0048856 | anatomical structure development | 31 |
| 18 | GO:0080090 | regulation of primary metabolic process | 30 |
| 19 | GO:0031323 | regulation of cellular metabolic process | 30 |
| 20 | GO:0019219 | regulation of nucleobase-containing compound metabolic process | 27 |
| 21 | GO:0051171 | regulation of nitrogen compound metabolic process | 27 |
| 22 | GO:0043412 | macromolecule modification | 26 |
| 23 | GO:0006464 | cellular protein modification process | 24 |
| 24 | GO:0036211 | protein modification process | 24 |
| 25 | GO:0016070 | RNA metabolic process | 23 |
| 26 | GO:0000003 | reproduction | 18 |
| 27 | GO:0008104 | protein localization | 17 |
| 28 | GO:0009790 | embryo development | 17 |
| 29 | GO:0033036 | macromolecule localization | 17 |
| 30 | GO:0022414 | reproductive process | 16 |
| 31 | GO:0009653 | anatomical structure morphogenesis | 16 |
| 32 | GO:0048519 | negative regulation of biological process | 15 |
| 33 | GO:0048583 | regulation of response to stimulus | 15 |
| 34 | GO:0048518 | positive regulation of biological process | 15 |
| 35 | GO:0048869 | cellular developmental process | 15 |
| 36 | GO:0010646 | regulation of cell communication | 14 |
| 37 | GO:0051641 | cellular localization | 14 |
| 38 | GO:0050790 | regulation of catalytic activity | 14 |
| 39 | GO:0065009 | regulation of molecular function | 14 |
| 40 | GO:0030154 | cell differentiation | 14 |
| 41 | GO:0048731 | system development | 14 |
| 42 | GO:0044702 | single organism reproductive process | 13 |
| 43 | GO:0009966 | regulation of signal transduction | 13 |
| 44 | GO:0051649 | establishment of localization in cell | 13 |
| 45 | GO:0023051 | regulation of signaling | 13 |
| 46 | GO:0003006 | developmental process involved in reproduction | 12 |
| 47 | GO:0072521 | purine-containing compound metabolic process | 12 |
| 48 | GO:1902531 | regulation of intracellular signal transduction | 11 |
| 49 | GO:0016192 | vesicle-mediated transport | 11 |
| 50 | GO:0035556 | intracellular signal transduction | 11 |
| 51 | GO:0048523 | negative regulation of cellular process | 11 |
| 52 | GO:0048522 | positive regulation of cellular process | 11 |
| 53 | GO:0034655 | nucleobase-containing compound catabolic process | 11 |
| 54 | GO:0019439 | aromatic compound catabolic process | 11 |
| 55 | GO:0044270 | cellular nitrogen compound catabolic process | 11 |
| 56 | GO:0046700 | heterocycle catabolic process | 11 |
| 57 | GO:1901361 | organic cyclic compound catabolic process | 11 |
| 58 | GO:0048598 | embryonic morphogenesis | 10 |
| 59 | GO:0022402 | cell cycle process | 10 |
| 60 | GO:0007049 | cell cycle | 10 |
| 61 | GO:0019220 | regulation of phosphate metabolic process | 10 |
| 62 | GO:0051174 | regulation of phosphorus metabolic process | 10 |

**Table S4.** **Gene ontology terms with gene counts more than 10 based on up-regulated miRNAs induced by *C. albicans* infection**

| No. | GO accession | GO term | Count |
| --- | --- | --- | --- |
| 1 | GO:0005623 | cell | 1301 |
| 2 | GO:0044464 | cell part | 1296 |
| 3 | GO:0005622 | intracellular | 1142 |
| 4 | GO:0044424 | intracellular part | 1119 |
| 5 | GO:0043226 | organelle | 942 |
| 6 | GO:0043229 | intracellular organelle | 931 |
| 7 | GO:0043227 | membrane-bounded organelle | 788 |
| 8 | GO:0043231 | intracellular membrane-bounded organelle | 770 |
| 9 | GO:0005737 | cytoplasm | 645 |
| 10 | GO:0005634 | nucleus | 519 |
| 11 | GO:0044422 | organelle part | 395 |
| 12 | GO:0044446 | intracellular organelle part | 365 |
| 13 | GO:0032991 | macromolecular complex | 363 |
| 14 | GO:0043234 | protein complex | 302 |
| 15 | GO:0044428 | nuclear part | 126 |
| 16 | GO:0031090 | organelle membrane | 122 |
| 17 | GO:0031974 | membrane-enclosed lumen | 103 |
| 18 | GO:0070013 | intracellular organelle lumen | 102 |
| 19 | GO:0043233 | organelle lumen | 102 |
| 20 | GO:0012505 | endomembrane system | 90 |
| 21 | GO:0044430 | cytoskeletal part | 89 |
| 22 | GO:0031981 | nuclear lumen | 88 |
| 23 | GO:0005783 | endoplasmic reticulum | 80 |
| 24 | GO:0005694 | chromosome | 77 |
| 25 | GO:0015630 | microtubule cytoskeleton | 57 |
| 26 | GO:0031410 | cytoplasmic vesicle | 53 |
| 27 | GO:0031982 | vesicle | 53 |
| 28 | GO:0044427 | chromosomal part | 52 |
| 29 | GO:0016023 | cytoplasmic membrane-bounded vesicle | 46 |
| 30 | GO:0031988 | membrane-bounded vesicle | 46 |
| 31 | GO:0044432 | endoplasmic reticulum part | 42 |
| 32 | GO:0005575 | cellular_component | 41 |
| 33 | GO:0042175 | nuclear outer membrane-endoplasmic reticulum membrane network | 38 |
| 34 | GO:0005789 | endoplasmic reticulum membrane | 37 |
| 35 | GO:0044431 | Golgi apparatus part | 35 |
| 36 | GO:0005819 | spindle | 28 |
| 37 | GO:0045177 | apical part of cell | 28 |
| 38 | GO:0035770 | ribonucleoprotein granule | 27 |
| 39 | GO:0000785 | chromatin | 25 |
| 40 | GO:0043186 | P granule | 24 |
| 41 | GO:0045495 | pole plasm | 24 |
| 42 | GO:0060293 | germ plasm | 24 |
| 43 | GO:0016324 | apical plasma membrane | 22 |
| 44 | GO:0030135 | coated vesicle | 22 |
| 45 | GO:0005768 | endosome | 20 |
| 46 | GO:0030139 | endocytic vesicle | 19 |
| 47 | GO:0005667 | transcription factor complex | 19 |
| 48 | GO:0044433 | cytoplasmic vesicle part | 15 |
| 49 | GO:0030117 | membrane coat | 14 |
| 50 | GO:0048475 | coated membrane | 14 |
| 51 | GO:0012506 | vesicle membrane | 14 |
| 52 | GO:0030659 | cytoplasmic vesicle membrane | 14 |
| 53 | GO:0045335 | phagocytic vesicle | 13 |
| 54 | GO:0005882 | intermediate filament | 11 |
| 55 | GO:0016591 | DNA-directed RNA polymerase II, holoenzyme | 11 |
| 56 | GO:0045111 | intermediate filament cytoskeleton | 11 |
| 57 | GO:0005769 | early endosome | 10 |
| 58 | GO:0016592 | mediator complex | 10 |

**Table S5. Signaling pathways with the gene number (matched with entitylist) based on KEGG assay for the down-regulated miRNAs induced by *C. albicans* infection**

| No. | Pathway | Count |
| --- | --- | --- |
| 1 | Metabolic pathways | 9 |
| 2 | Endocytosis | 6 |
| 3 | ErbB signaling pathway | 5 |
| 4 | MAPK signaling pathway | 4 |
| 5 | Wnt signaling pathway | 4 |
| 6 | Spliceosome | 4 |
| 7 | Protein processing in endoplasmic reticulum | 4 |
| 8 | Jak-STAT signaling pathway | 3 |
| 9 | TGF-beta signaling pathway | 3 |
| 10 | Phagosome | 3 |
| 11 | Ubiquitin mediated proteolysis | 3 |
| 12 | Natural killer cell mediated cytotoxicity | 2 |
| 13 | Progesterone-mediated oocyte maturation | 2 |
| 14 | Arginine and proline metabolism | 2 |
| 15 | Calcium signaling pathway | 2 |
| 16 | mRNA surveillance pathway | 2 |
| 17 | Ribosome biogenesis in eukaryotes | 2 |
| 18 | Lysosome | 2 |
| 19 | Purine metabolism | 2 |
| 20 | Oxidative phosphorylation | 2 |
| 21 | Mucin type O-Glycan biosynthesis | 1 |
| 22 | Dorso-ventral axis formation | 1 |
| 23 | Hedgehog signaling pathway | 1 |
| 24 | Retinol metabolism | 1 |
| 25 | Homologous recombination | 1 |
| 26 | Mismatch repair | 1 |
| 27 | Notch signaling pathway | 1 |
| 28 | Inositol phosphate metabolism | 1 |
| 29 | RNA polymerase | 1 |
| 30 | Circadian rhythm - mammal | 1 |
| 31 | mTOR signaling pathway | 1 |
| 32 | Basal transcription factors | 1 |
| 33 | Phosphatidylinositol signaling system | 1 |
| 34 | DNA replication | 1 |
| 35 | Nucleotide excision repair | 1 |
| 36 | N-Glycan biosynthesis | 1 |
| 37 | Proteasome | 1 |
| 38 | Valine, leucine and isoleucine degradation | 1 |
| 39 | Peroxisome | 1 |
| 40 | Pyrimidine metabolism | 1 |
| 41 | RNA transport | 1 |

**Table S6. Signaling pathways with the gene number (matched with entitylist) based on KEGG assay for the up-regulated miRNAs induced by *C. albicans* infection**

| No. | Pathway | Count |
| --- | --- | --- |
| 1 | Metabolic pathways | 149 |
| 2 | Protein processing in endoplasmic reticulum | 43 |
| 3 | Wnt signaling pathway | 40 |
| 4 | Endocytosis | 39 |
| 5 | MAPK signaling pathway | 37 |
| 6 | Ubiquitin mediated proteolysis | 37 |
| 7 | Purine metabolism | 33 |
| 8 | RNA transport | 33 |
| 9 | Oxidative phosphorylation | 27 |
| 10 | Calcium signaling pathway | 24 |
| 11 | Phagosome | 24 |
| 12 | Spliceosome | 23 |
| 13 | ErbB signaling pathway | 22 |
| 14 | Progesterone-mediated oocyte maturation | 22 |
| 15 | Lysosome | 22 |
| 16 | mRNA surveillance pathway | 20 |
| 17 | Fatty acid metabolism | 18 |
| 18 | Ribosome biogenesis in eukaryotes | 18 |
| 19 | Pyrimidine metabolism | 17 |
| 20 | Phosphatidylinositol signaling system | 14 |
| 21 | TGF-beta signaling pathway | 14 |
| 22 | Nucleotide excision repair | 14 |
| 23 | Glycerophospholipid metabolism | 14 |
| 24 | Valine, leucine and isoleucine degradation | 14 |
| 25 | Peroxisome | 14 |
| 26 | Natural killer cell mediated cytotoxicity | 13 |
| 27 | mTOR signaling pathway | 13 |
| 28 | Amino sugar and nucleotide sugar metabolism | 13 |
| 29 | DNA replication | 13 |
| 30 | Proteasome | 12 |
| 31 | Basal transcription factors | 11 |
| 32 | Propanoate metabolism | 11 |
| 33 | Lysine degradation | 11 |
| 34 | RNA degradation | 11 |
| 35 | Inositol phosphate metabolism | 10 |
| 36 | Circadian rhythm - mammal | 10 |
| 37 | Neuroactive ligand-receptor interaction | 10 |
| 38 | Ribosome | 10 |
| 39 | Glutathione metabolism | 9 |
| 40 | Dorso-ventral axis formation | 8 |
| 41 | Hedgehog signaling pathway | 8 |
| 42 | Mismatch repair | 8 |
| 43 | Notch signaling pathway | 8 |
| 44 | Glycerolipid metabolism | 8 |
| 45 | Starch and sucrose metabolism | 8 |
| 46 | Tryptophan metabolism | 8 |
| 47 | Glycolysis / Gluconeogenesis | 8 |
| 48 | Jak-STAT signaling pathway | 7 |
| 49 | Fructose and mannose metabolism | 7 |
| 50 | Butanoate metabolism | 7 |
| 51 | Alanine, aspartate and glutamate metabolism | 7 |
| 52 | N-Glycan biosynthesis | 7 |
| 53 | Arginine and proline metabolism | 7 |
| 54 | Base excision repair | 6 |
| 55 | Protein export | 6 |
| 56 | Metabolism of xenobiotics by cytochrome P450 | 6 |
| 57 | Drug metabolism - cytochrome P450 | 6 |
| 58 | Glycosaminoglycan biosynthesis - heparan sulfate | 5 |
| 59 | Homologous recombination | 5 |
| 60 | Limonene and pinene degradation | 5 |
| 61 | beta-Alanine metabolism | 5 |
| 62 | Tyrosine metabolism | 5 |
| 63 | Sphingolipid metabolism | 5 |
| 64 | Pyruvate metabolism | 5 |
| 65 | Drug metabolism - other enzymes | 5 |
| 66 | Nicotinate and nicotinamide metabolism | 4 |
| 67 | Regulation of autophagy | 4 |
| 68 | Ubiquinone and other terpenoid-quinone biosynthesis | 4 |
| 69 | Arachidonic acid metabolism | 4 |
| 70 | Glyoxylate and dicarboxylate metabolism | 4 |
| 71 | Retinol metabolism | 4 |
| 72 | Pentose phosphate pathway | 4 |
| 73 | Nitrogen metabolism | 4 |
| 74 | RNA polymerase | 4 |
| 75 | ABC transporters | 3 |
| 76 | Non-homologous end-joining | 3 |
| 77 | Glycosaminoglycan biosynthesis - chondroitin sulfate | 3 |
| 78 | Mucin type O-Glycan biosynthesis | 3 |
| 79 | ECM-receptor interaction | 3 |
| 80 | Ether lipid metabolism | 3 |
| 81 | Terpenoid backbone biosynthesis | 3 |
| 82 | Galactose metabolism | 3 |
| 83 | Fatty acid elongation in mitochondria | 3 |
| 84 | Other glycan degradation | 3 |
| 85 | Other types of O-glycan biosynthesis | 3 |
| 86 | Biosynthesis of unsaturated fatty acids | 3 |
| 87 | Ascorbate and aldarate metabolism | 3 |
| 88 | Phenylalanine metabolism | 3 |
| 89 | Pentose and glucuronate interconversions | 3 |
| 90 | Citrate cycle (TCA cycle) | 3 |
| 91 | One carbon pool by folate | 2 |
| 92 | Selenocompound metabolism | 2 |
| 93 | Histidine metabolism | 2 |
| 94 | Porphyrin and chlorophyll metabolism | 2 |
| 95 | Glycine, serine and threonine metabolism | 2 |
| 96 | SNARE interactions in vesicular transport | 2 |
| 97 | Cysteine and methionine metabolism | 2 |
| 98 | Fatty acid biosynthesis | 1 |
| 99 | Polyketide sugar unit biosynthesis | 1 |
| 100 | Glycosaminoglycan biosynthesis - keratan sulfate | 1 |
| 101 | Glycosphingolipid biosynthesis - ganglio series | 1 |
| 102 | Synthesis and degradation of ketone bodies | 1 |
| 103 | Folate biosynthesis | 1 |
| 104 | Phenylalanine, tyrosine and tryptophan biosynthesis | 1 |
| 105 | Riboflavin metabolism | 1 |
| 106 | Glycosylphosphatidylinositol(GPI)-anchor biosynthesis | 1 |
| 107 | Sulfur relay system | 1 |
| 108 | alpha-Linolenic acid metabolism | 1 |

**Table S7.** **Primers for reverse transcription of miRNAs**

| miRNA | Primer |
| --- | --- |
| *mir-240* | GTCGTATCCAGTGCAGGGTCCGAGGTATTCGCACTGGATACGACAGCGAA |
| *mir-75* | GTCGTATCCAGTGCAGGGTCCGAGGTATTCGCACTGGATACGACTGAAGC |
| *mir-62* | GTCGTATCCAGTGCAGGGTCCGAGGTATTCGCACTGGATACGACCTGTAA |
| *mir-251* | GTCGTATCCAGTGCAGGGTCCGAGGTATTCGCACTGGATACGACTAAGAG |
| *mir-252* | GTCGTATCCAGTGCAGGGTCCGAGGTATTCGCACTGGATACGACTTACCT |
| *mir-1821* | GTCGTATCCAGTGCAGGGTCCGAGGTATTCGCACTGGATACGACTCTACC |
| *mir-254* | GTCGTATCCAGTGCAGGGTCCGAGGTATTCGCACTGGATACGACGTCGCG |
| *mir-53* | GTCGTATCCAGTGCAGGGTCCGAGGTATTCGCACTGGATACGACAGCACG |
| *mir-86* | GTCGTATCCAGTGCAGGGTCCGAGGTATTCGCACTGGATACGACGACTGT |
| *mir-360* | GTCGTATCCAGTGCAGGGTCCGAGGTATTCGCACTGGATACGACTGACCGT |

**Table S8. Primers for real-time PCR of miRNAs**

| miRNA | primer |
| --- | --- |
| *mir-240* | GTACTGGCCCCCAAATCTTCG |
| *mir-75* | TAAAGCTACCAACCGGCTTCA |
| *mir-62* | TGATATGTAATCTAGCTTACAG |
| *mir-251* | GGTTAAGTAGTGGTGCCGCTC |
| *mir-252* | ATAAGTAGTAGTGCCGCAGG |
| *mir-1821* | TGAGGTCTTATAGTTAGGTAG |
| *mir-254* | GGTGCAAATCTTTCGCGAC |
| *mir-53* | GCACCCGTACATTTGTTTCCG |
| *mir-86* | TAAGTGAATGCTTTGCCACAG |
| *mir-360* | TGCTACTTGTGACCGTTGTTAC |
| Common reward primer | GTGCAGGGTCCGAGGT |
| *F35C11.9*/forward primer | GAAGATTAGCATGAACCC |
| *F35C11.9*/reverse primer | TTGGAACGCTTTATGAAT |

**Table S9.** **Primers for real-time PCR of antimicrobial genes**

| Gene | Forward primer | Reverse primer |
| --- | --- | --- |
| *abf-2* | TGGTAATGCACAACCCCTGA | TTCGTCCGTTCCCTTTTCCT |
| *cnc-4* | GCTTCGCTACATTCTCGTCCT | GTATCCACCACCATACCCGC |
| *cnc-7* | GGACGGTACATTCCCATACC | CAGGTTCAATGCAGTATGGCTATGG |
| *fipr-22/23* | GCTGAAGCTCCACACATCC | TATCCCATTCCTCCGTATCC |
| *act-1* | CTGCAGATGTGTGACGACGAGGTT | CTGCAGGAAGCACTTGCGGTGAAC |
